# Shell Phase and Morphology Control for Emission Tuning in III–V Core/Shell Quantum Dots

**DOI:** 10.1021/acsnano.5c10168

**Published:** 2025-08-05

**Authors:** Xiang Li, Einav Scharf, Adar Levi, Yinon Deree, David Stone, Sergei Remennik, Uri Banin

**Affiliations:** The Institute of Chemistry and The Center for Nanoscience and Nanotechnology, 98519 The Hebrew University of Jerusalem, Jerusalem 91904, Israel

**Keywords:** III−V semiconductors, epitaxial growth, shell morphology control, Ostwald ripening, crystal phase transformation

## Abstract

Epitaxial growth of shells on III–V semiconductor quantum dot (QD) cores yields improved fluorescence quantum efficiency and stability toward their implementation in light emission technologies. Here, we control the shell morphology and crystal structure and investigate their effects on the emission properties of heterovalent III–V/II–VI core/shell QDs. This is achieved by tuning the ZnSe shell growth mode from kinetic to thermodynamic regimes via adjusting the precursor reactivity. When combined with high-temperature Ostwald ripening, this approach enables controlled tuning of shell morphology between tetrahedral and spherical-like, accompanied by a transformation of the shell crystal structure from zinc-blende to wurtzite. The position of the III–V cores within the III–V/ZnSe core/shell QDs varies under the different growth modes, being closer to the edge in the former. Moreover, the spherical architecture exhibits a higher photoluminescence quantum yield (PLQY) and improved stability. Such morphological and crystal-type differences directly affect the band alignment and exciton confinement, leading to tunable emission spectra and exciton dynamics, as confirmed by quantum mechanical simulations of the band gap exciton energies. This study deepens the understanding of heteroepitaxial growth and emission control in III–V/II–VI core/shell QDs, enabling advanced QD design toward optimization for diverse light emission applications.

## Graphical abstract

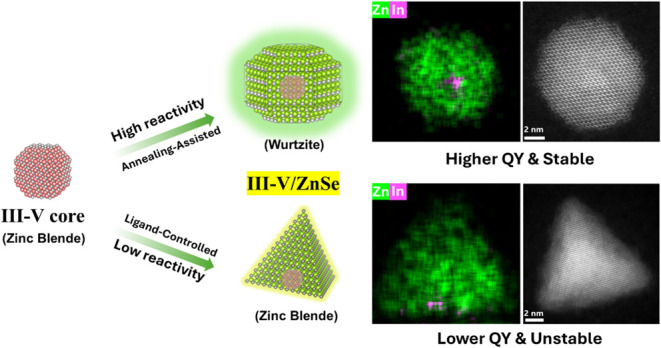

## Introduction

Colloidal semiconductor quantum dots (QDs), which exhibit size-dependent optoelectronic properties due to the quantum confinement effect,^1,2^ have been intensively studied and have reached the break point of commercialization in numerous fields such as display and lighting, quantum information, solar energy conversion, and biological sensors.^3–7^ Over the past two decades, advances in synthetic techniques have allowed for precise control over the size, shape, and composition of nanocrystals, resulting in QDs with highly tuned and well-defined characteristics.^8,9^ However, most research efforts are devoted to cadmium- and lead-based QDs, and it is the toxicity and regulatory constraints on these heavy metal-containing QDs that limit their applications.^10^ Among the various binary QDs that have been explored, III–V QDs show excellent development opportunities due to their low toxicity, wide emission ranges tunable from the visible to the near-infrared regions, and additional favorable optoelectronic properties.^11,12^ Among them, indium phosphide (InP) and indium arsenide (InAs) quantum dots have attracted significant interest. Advances in epitaxial shell growth have greatly enhanced their photoluminescence quantum yield (PLQY) and stability, enabling applications in displays and light-emitting diodes (LEDs).^13,14^ Additionally, water-soluble InP-based QDs developed via surface copassivation strategies exhibit excellent performance in bioimaging.^15^ These developments underscore their promise as environmentally friendly, high-performance candidates for next-generation colloidal QD technologies.

As QDs typically possess a large specific surface area, their optical and optoelectronic properties are highly susceptible to surface trap states.^16,17^ Therefore, surface passivation is crucial for achieving strongly luminescent and stable QDs. Epitaxial growth of protective semiconductor shells on QD cores is the most common strategy for passivating QD surface defects.^18,19^ For instance, numerous studies have demonstrated that the epitaxial shell of ZnSe on the surface of III–V QDs effectively confines both electrons and holes within the core by forming a type-I straddling band alignment, thereby reducing nonradiative recombination through the passivation of surface trap sites and improving the luminescence efficiency and stability of QDs.^20–23^

Recent research has highlighted the adverse effect of surface oxidation of III–V QDs on the structural uniformity of the epilayers. Fluorides such as hydrofluoric acid (HF) were used to etch away the InPO_*x*_ oxidation layer prior to shell growth, thereby increasing the quantum yield of InP QDs with more uniform morphology.^22,24–26^ However, the use of fluoride has safety risks, and the amount of HF is difficult to precisely control, which often leads to broadening of the size distribution and also to overetching of the InP core and the generation of shallow hole traps.^27^ In addition, adjusting the precursor reactivity and reaction temperature is also a crucial parameter for improving the morphological uniformity of the epitaxial layer.^22,28–30^ Despite significant progress, the precise control of morphology and crystalline phase of heteroepitaxial layers on III–V QD surfaces still requires an additional deep understanding.

In this study, we introduce a straightforward strategy to tune the shape and crystalline phase of III–V/ZnSe core–shell QDs by controlling the shell precursor reactivity. When trioctylphosphine (TOP) was added to the reaction system, it reduced the reactivity of the Se precursor. In the presence of such low-reactivity precursors, the ZnSe shell growth is biased toward the formation of thermodynamically stable [111] facets, leading to tetrahedral III–V/ZnSe core/shell QDs with both the core and shell exhibiting a zinc-blende (ZB) structure. Under the high-reactivity conditions without TOP, the kinetically controlled growth mode is dominant. With the assistance of Ostwald ripening at high temperature, isotropic shell growth can be achieved, resulting in the shell morphology becoming spherical, while the shell crystal structure also changes from ZB to wurtzite (WZ).

A comparative analysis of the optical properties shows that the spherical-WZ structure achieves a better passivation and protection effect on the III–V core QDs, significantly improving the PLQY and stability compared with the tetrahedral counterpart. Interestingly, the core position in core–shell QDs under different growth modes further affects the tunability of their emission spectra and exciton dynamics. Quantum mechanical simulations were used to calculate the exciton band gap energies directly, revealing the sensitivity to varying the band offset between the core and shell regions, in line with the expected trend of the energetics between ZB and WZ morphologies. This work, combining experimental and theoretical approaches, extends the control of the photophysical properties of III–V core/shell QDs. It provides a general strategy for obtaining high-quality III–V semiconductor-based core/shell QDs with improved characteristics, offering significant potential for optoelectronic and bioimaging applications.

## Results and Discussion

In order to comprehensively compare InP/ZnSe core/shell QDs with different crystal forms and morphologies, we first prepared the InP core according to the established hot-injection method.^31,32^ As shown in (Figure S1a, Supporting Information), the InP core exhibits a sharp first excitonic transition peak at 450 nm, indicating a narrow size distribution, while its photoluminescence (PL) is very weak due to surface traps. The average diameter of the InP cores is ∼2.9 nm, and the X-ray powder diffraction (XRD) pattern is consistent with the zinc-blende crystal structure (Figure S1b,c, Supporting Information).^22^ Further, ZnSe epilayers were selected to grow on the InP core, forming a type-I heterogeneous core/shell structure to improve its fluorescence efficiency and stability.^19,22^ The detailed synthetic description is provided in the Supporting Information. Two distinct synthesis strategies were implemented to prepare the InP/ZnSe core/shell QDs, as illustrated in Figure 1a. Aliquots of the InP QDs stock solution were used as seeds for the growth of the ZnSe shell. The precursors utilized for the ZnSe shell growth were zinc stearate and selenium powder suspensions. However, we found that the shell growth process can be tuned to form either a tetrahedral-ZB or spherical-WZ-ZnSe shell, depending on the difference in reactivity caused by the presence of TOP in the precursor (Figure 1a).

**Figure 1:**
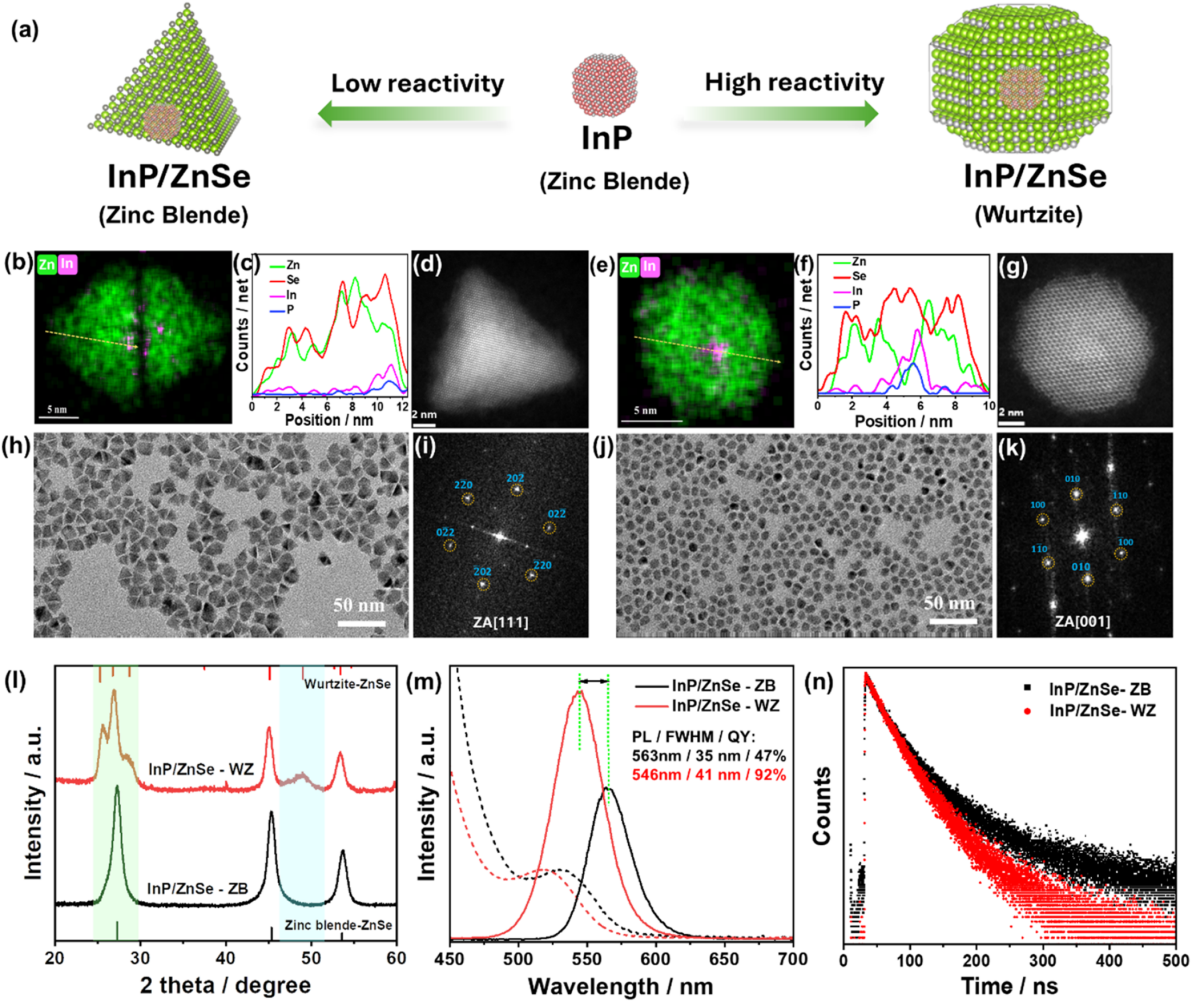
(a) Schematics illustrating the morphology control of ZnSe epilayers grown on InP QDs. (b, e) EDS elemental mapping, (c, f) EDS line scan, (d, g) HAADF-STEM image, (h, j) TEM images, (i, k) Corresponding fast Fourier transform patterns, (l) XRD patterns, with the standard diffraction peaks of WZ-ZnSe (JCPDS No. 80–0008) and ZB-ZnSe (JCPDS No. 37–1463) shown at the top and bottom of the plot, respectively, (m) UV–vis absorption and PL spectra, and (n) PL decay traces of InP/ZnSe-ZB and InP/ZnSe-WZ QDs.

For the deposition of the ZnSe shell, the ZB InP cores were introduced into a mixture of zinc stearate in squalane and TOP, and the shell growth process was conducted at 300–340 °C. The transmission electron microscopy (TEM) image in Figure 1h shows that a tetrahedral InP/ZnSe (InP/ZnSe-ZB) core/shell structure is obtained, with a volume of approximately 400 nm^3^ (Figure S2, Supporting Information). The high-angle annular dark-field-scanning TEM (HAADF-STEM) image manifests the (220) lattice plane, typical of zinc-blende ZnSe structure,^35^ with continuous atomic lattice fringes throughout the whole QD and no obvious stacking faults (Figure 1d). Fast Fourier Transform (FFT) images (Figure 1i) acquired from the HAADF-STEM image correspond to the view along the [111] zone axis (ZA).^36^ Interestingly, the elemental mapping via energy-dispersive X-ray spectroscopy (EDS) reveals the positions that the InP core is typically off-centered, closer to the edge of the QDs (Figures 1b and S3–S4, Supporting Information). This is further confirmed by an EDS line scan measurement (Figure 1c). However, if TOP is not added during the shell growth process but a high-temperature ripening-assisted operation is introduced, then a spherical InP/ZnSe (InP/ZnSe-WZ) core/shell structure can eventually be obtained (Figure 1j). Figure 1g,k shows HAADF-STEM and FFT images of InP/ZnSe-WZ QDs, viewed under [001] ZA of their wurtzite structure.^36,37^ Furthermore, the EDS elemental map clearly shows that the InP core tends to be in the center of the ZnSe shell (Figures 1e,f and S5, Supporting Information). Interestingly, such off-center core positioning is not unique to the present III–V system. Similar asymmetric geometries were also observed in our earlier study on CdSe/CdS tetrahedral heterostructures, although their optical implications were not examined at the time.^38^ The recurrence of this structural feature across distinct material systems suggests that the off-center configuration may represent a more general and potentially underexplored structural motif in tetrahedral core/shell QDs. Its influence on carrier localization, exciton dynamics, and directional energy transfer may warrant broader attention. In this work, we further investigate in the following sections how such asymmetric structures affect the photoluminescence properties and stability.

The XRD patterns confirm that the ZnSe shells, grown epitaxially on the same ZB InP core under the different conditions, manifest two different crystal structures (Figure 1l). In tetrahedral InP/ZnSe-ZB, the ZnSe shell retains the same zinc-blende crystal structure as the InP core, whereas the ZnSe shell in spherical InP/ZnSe-WZ primarily exhibits a wurtzite crystal structure, differing from the crystal structure of the InP core.^22,37^ In order to identify the InP/ZnSe core/shell structures, we investigated the atomic resolution HRTEM images, followed by FFT analysis and the corresponding simulated structure model. Figure S6a–d (Supporting Information) presents tetrahedral InP/ZnSe-ZB QDs observed under ZA of [111], [100], [110], and [112̅], respectively, while Figure S6e,f (Supporting Information) depicts spherical InP/ZnSe-WZ QDs viewed along the ZA of [110] and [001], respectively. The characterization results confirm that the synthesized InP/ZnSe-ZB QDs exhibit a tetrahedral shape consistent with the ZB crystal structure, whereas the InP/ZnSe-WZ QDs display a spherical shape corresponding to the WZ crystal structure. Generally, the crystal structure of the core determines the crystalline phase of the shell, while ligands always play a crucial role in controlling morphology and determining the phase structure during crystal growth.^39,40^ We compared the reactivity of the precursors in the two synthetic strategies via a hot-injection method at 300 and 340 °C. By monitoring the appearance of the exciton absorption peak in the ultraviolet visible (UV–vis) absorption spectrum and the change in absorbance at 300 nm over time, it can be determined that the introduction of TOP significantly reduces the reaction rate of Se (Figure S7, Supporting Information).^41,42^ Considering that TOP serves as a ligand that selectively passivates the InP surface facets, it is understandable that the final crystal form and morphology remain consistent with the InP core.^43–45^ A possible reason for the eccentric positioning of the InP core within the ZnSe shell may be the different growth rates of the formed ZB shell layers.

Notably, we observed differences in the fluorescence properties resulting from variations in shell growth. Figure 1m,n presents a comparison between the optical properties of InP cores coated by tetrahedral or spherical ZnSe shells of similar volume (V_ZnSe_ ≈ 400 nm^3^). Interestingly, the PL spectrum of spherical InP/ZnSe-WZ QDs displayed a blue shift and an increase in the absolute PLQY (546 nm and 92%, respectively) compared to the tetrahedral InP/ZnSe-ZB QDs (563 nm and 47%, respectively). To gain deeper insight into the radiative and nonradiative recombination dynamics of InP/ZnSe QDs, we performed time-resolved photoluminescence (TRPL) measurements. The PL decay curves were fitted using a biexponential function, and the results revealed that InP/ZnSe-WZ QDs exhibit a slightly shorter average lifetime (89 ns) compared to InP/ZnSe-ZB QDs (100 ns). By combining the average lifetime with absolute PLQY measurements, the radiative (*k*_rad_) and nonradiative (*k*_nrad_) recombination rate constants for both QD types were extracted (Table S1, Supporting Information).^46^ Typically, InP/ZnSe-WZ QDs demonstrate a higher radiative recombination rate and a lower nonradiative recombination rate relative to InP/ZnSe-ZB QDs, consistent with their higher PLQY. These findings suggest that InP/ZnSe-WZ QDs exhibit a more efficient emission process due to reduced nonradiative losses. We attribute this difference primarily to the spatial configuration of the InP core within the core/shell structure. Specifically, in the InP/ZnSe-ZB QDs, the InP core tends to be positioned closer to the outer surface, resulting in suboptimal surface passivation and increased exposure to surface trap states. This core–shell misalignment may facilitate nonradiative recombination pathways. A more comprehensive comparative analysis will be presented in the following sections.

Thus far, related heterocrystalline (ZB/WZ) core/shell QDs with III–V cores were limited to Cd-containing shells, such as InP/CdS,^47^ InP/CdSe,^48^ and InP/ZnCdSe.^49^ To understand the different growth patterns of the InP/ZnSe heterocrystalline shell, we studied the mechanism of the shell growth in the absence of TOP. Here, we observed that when the reaction temperature was set to 300 °C, a multibranched core/shell structure resembling a tetrapod (InP/ZnSe tetrapod) was obtained (Figures S8a and S9, Supporting Information). Upon increasing the temperature to 340 °C, the morphology transformed into spherical-like InP/ZnSe nanostructures with uniform shells. TEM analysis revealed that the ZnSe shell grown at higher temperatures exhibited improved uniformity and sphericity (Figure S8b,c, Supporting Information). The optical properties of InP/ZnSe core/shell quantum dots synthesized at three different temperature settings were compared (Figure S8d,e and Table S2, Supporting Information). Notably, gradient heating resulted in InP/ZnSe QDs with higher PLQY and narrower fluorescence peak widths. This improvement may be attributed to the gradient heating process mitigating Ostwald ripening of the InP core that takes place at elevated temperatures.^50^ Furthermore, XRD analysis provided intriguing insights: the InP/ZnSe tetrapod structure obtained at lower temperatures exhibited a crystal structure consistent with the zinc-blende phase, whereas higher temperatures favored the formation of the wurtzite phase (Figure S8f, Supporting Information).

Building on the aforementioned findings, we explored and designed a three-step synthesis scheme for the synthesis of InP/ZnSe-WZ core–shell QDs (Figure 2a). In general, the as-obtained InP cores were injected into the zinc stearate and squalane solution. In the first step, selenium suspension (Se-SUS) was slowly dropped into the flask at 300 °C and kept for 30 min, during which a thin ZnSe layer was initially formed on the InP core. This preliminary shell serves as a protective barrier to minimize core degradation or ripening during the subsequent high-temperature shell growth.^13^ The morphology of the as-obtained QDs is hard to control, and short InP/ZnSe tetrapods are observed in Figure 2b. These represent an intermediate state during shell growth. With an extended reaction time at 300 °C, more defined tetrapod structures emerge, but overly prolonged growth can hinder subsequent shape evolution (Figure S10, Supporting Information). Thus, we chose a 30 min reaction to capture this transitional morphology. Then, the reaction temperature was raised to 340 °C for 30 min in the second step. High-temperature thermal annealing is used to form InP/ZnSe QDs into a thermodynamically favored spherical shape (Figure 2c). In the third step, a temperature of 340 °C was maintained. Se-SUS was again added dropwise to the flask, and the reaction resulted in the final InP/ZnSe-WZ QDs. Figure 2d confirms that the resulting InP/ZnSe-WZ QDs remained nearly spherical in shape along the epitaxial growth. Through the annealing-assisted operation, not only a controllable change in morphology was achieved, but also a crystalline change from zinc-blende to wurtzite was discovered. The HAADF-STEM images and the corresponding FFT images of InP/ZnSe tetrapod and spherical InP/ZnSe clearly show [111] and [002] zinc-blende crystal lattice planes at the [112] and [110] zone axes, respectively (Figure 2e,f and h,i). In contrast, the final InP/ZnSe-WZ QDs show [100] and [010] wurtzite lattice planes on the [001] zone axes (Figure 2g,j).

**Figure 2:**
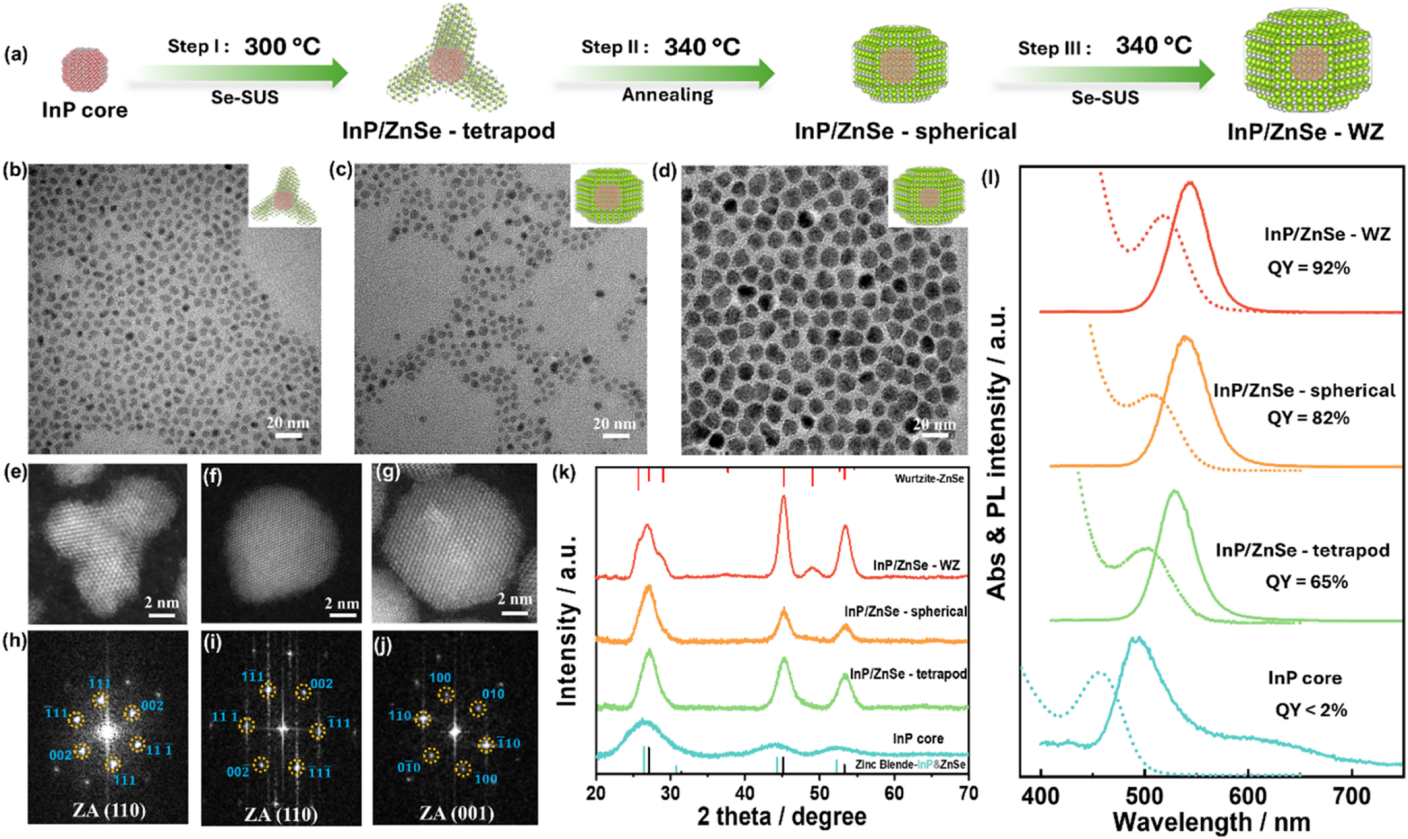
(a) Schematics of the synthesis of heterophased InP/ZnSe-WZ core–shell QDs. (b–d) TEM images, (e–g) HAADF-STEM, (h–j) FFT images, (k) XRD patterns, and (l) UV–vis absorption (dashed line) and PL spectra (solid line) at different points in the shell growth process of InP/ZnSe-WZ core–shell QDs.

To optimize the annealing time, we compared the morphology, crystal form, and optical properties of samples obtained at different times. According to the TEM images (Figure S11, Supporting Information), the size distribution of InP/ZnSe QDs slowly broadens as the annealing time increases. This typical change confirms the occurrence of Ostwald ripening. The XRD pattern (Figure S12, Supporting Information) shows that the crystal structure of InP/ZnSe QDs changes from zinc-blende to wurtzite. For ZnSe, it is reported that the zinc-blende phase is the thermodynamically stable state, while the wurtzite crystal structure is its metastable state.^51,52^ Previous studies have demonstrated that the phase transition of ZnSe from zinc-blende to wurtzite can be achieved by raising the temperature above 240 °C, thereby surpassing the associated energy barrier.^52^ This could help explain the changes in the crystal structure observed during annealing in our experiments. As the annealing time increases, both the UV–vis absorption spectrum and the PL spectrum exhibit a red shift, consistent with the change in size (Figure S13a, Supporting Information). Meanwhile, by comparing the fluorescence decay curve and absolute PLQY, we found that the average fluorescence lifetime first decreased and then increased, and the absolute PLQY peaked at 30 min (Figure S13b and Table S3, Supporting Information). Consequently, we opted to anneal for 30 min at 340 °C before proceeding to grow ZnSe shells of varying thicknesses. The evolution of XRD patterns, UV absorption, and PL spectra is shown in Figure 2k,l, respectively. The XRD pattern confirmed that indeed the resulting spherical shape, revealed by HRTEM, was accompanied by a change to wurtzite crystal structure. With increased shell thickness, both the fluorescence and absorption spectra exhibited a red shift, culminating in a final quantum yield of 92%. Interestingly, it has been established that after annealing, the InP/ZnSe tetrapods undergo a morphological transformation into a quasi-spherical shape, while maintaining the zinc-blende crystal structure. Given that extended reaction times at 340 °C led to phase transitions, we explored alternative growth conditions by continuing the shell growth at 300 °C following the annealing, with the aim of preserving crystal-invariant shell growth on spherical seeds. However, TEM analysis revealed that the final structure retained a multipod morphology. While XRD confirmed that the original crystal structure was preserved, PLQY decreased as the reaction time was extended (Figure S14, Supporting Information).

The shape evolution of InP/ZnSe core/shell nanocrystals is attributed to the complex interplay among facet-specific surface energies, precursor reactivity, and the balance between thermodynamic and kinetic growth mechanisms. In the zinc-blende structure, the surface energies of facets generally follow the order of γ [100] > γ [111] > γ [110], but this order may vary depending on the passivated facets and surface termination.^24,53^ Among these, the [111] facets, characterized by high atomic packing density and low dangling bond density, are typically more stable and thus preferentially preserved under thermodynamically controlled growth conditions. When the reactivity of the Se precursor is reduced by the addition of excess TOP, the growth rate of the ZnSe shell decreases significantly (Figure S7, Supporting Information). This slower deposition allows surface atoms to diffuse and reorganize, thereby minimizing the overall surface energy and favoring the expression of low-energy [111] facets. As a result, the growth mode is biased toward the thermodynamically favorable facets, leading to the formation of tetrahedral InP/ZnSe-ZB core/shell QDs enclosed by four [111] facets. Under such slow-growth conditions, however, the reduced deposition rate not only favors thermodynamic facet selection but also renders the relatively small InP core more susceptible to external perturbations during shell formation. In particular, lattice mismatch between the InP core and ZnSe shell may lead to asymmetric strain distribution at the core/shell interface, making an off-center positioning of the core energetically favorable.^54^

In the absence of TOP during the reaction, the Se precursor exhibits higher reactivity, resulting in a rapid shell growth governed by a kinetically controlled regime. Under these conditions, the growth rate surpasses the rate of surface atomic rearrangement, leading to an anisotropic shell extension along crystallographically favorable directions. Specifically, the [111] direction facilitates rapid atomic incorporation and crystal growth due to its polarity (Zn-rich) and high bonding density. This results in the formation of four protruding arms along the [111] direction, giving rise to a tetrapod-like nanostructure.^53^ This symmetric extension helps to maintain the InP core at the center of the growing shell by counterbalancing lateral displacements. Subsequently, high-temperature Ostwald ripening promotes surface reconstruction and migration of ZnSe atoms, enabling transformation into a more thermodynamically stable spherical ZB structure. This process has the potential to repair structural defects generated during the rapid growth stage, thereby enhancing the PLQY.^55,56^ Maintaining a higher temperature while continuously introducing highly reactive precursors sustains rapid shell growth. XRD patterns indicate a gradual transition of the shell from the ZB to WZ crystal structure. However, TEM images reveal that the particles maintain a quasi-spherical morphology, suggesting coherent and uniform crystal growth. Thus, this structural evolution may be attributed to the similar lattice parameters of the ZB and WZ structures along certain crystallographic directions, leading to a phase transition at high temperatures via localized stacking faults.^70^ This mechanism may facilitate the partial release of interface strain, which is consistent with the high PLQY and band-edge emission characteristics observed during shell growth.^28,57^

The tunability of optical properties, particularly fluorescence emission, is regarded as a distinctive advantage of semiconductor nanocrystals. We further compared the optical properties of the spherical and tetrahedral InP/ZnSe core/shell structures. Figure 3a presents a comparison of the PLQY of InP/ZnSe-ZB and InP/ZnSe-WZ QDs. As the volume of the ZnSe shell increases, the PLQY of InP/ZnSe core/shell QDs initially increases and then decreases. The maximum PLQY of InP/ZnSe-WZ QDs can reach approximately 90%, whereas the maximum PLQY of InP/ZnSe-ZB only reaches about 60%. The higher PLQY of the InP/ZnSe-WZ QDs can be attributed to the better passivation and protection by the spherical shell, where the core is at its center. A comparative analysis of the fluorescence spectra reveals that InP/ZnSe-ZB QDs exhibit a relatively narrower spectrum (Figure 3b). Additionally, following the PL peaks of the two types of QDs as the volume of the shell increases reveals that the two structures exhibit different extents of red shift. For the same InP core, growth of wurtzite shell eventually results in the PL peak at approximately 543 nm, whereas growth of zinc-blende shell results in the PL peak positioned around 560 nm (Figure 3b). This discrepancy in red shift may be related to the different band offset effects between the InP core and ZnSe shells with different crystal structures, which affect electron delocalization and exciton energy. We present simulation calculations to further elucidate this phenomenon. In addition, temperature-dependent PL measurements of InP/ZnSe-ZB and InP/ZnSe-WZ QDs were carried out to gain insight into the effect of the ZnSe shell on the emission. For these two samples, as the temperature increased from 77 to 180 K, a red shift of the emission peak can be observed, resulting from the band gap shrinkage and electron–phonon coupling effects known for bulk InP as well.^31,58–60^ Notably, for InP/ZnSe-ZB QDs, the decrease in temperature is accompanied by the evolution of a broad peak in the longer wavelength region (600–700 nm). We associate this with emission from trap states.^58,61^ This is not observed in the InP/ZnSe-WZ QDs (Figure 3c,d), providing further indication that the defects at the interface of the InP cores in InP/ZnSe-WZ QDs are more effectively passivated, which contributes to the improved PLQY.

**Figure 3:**
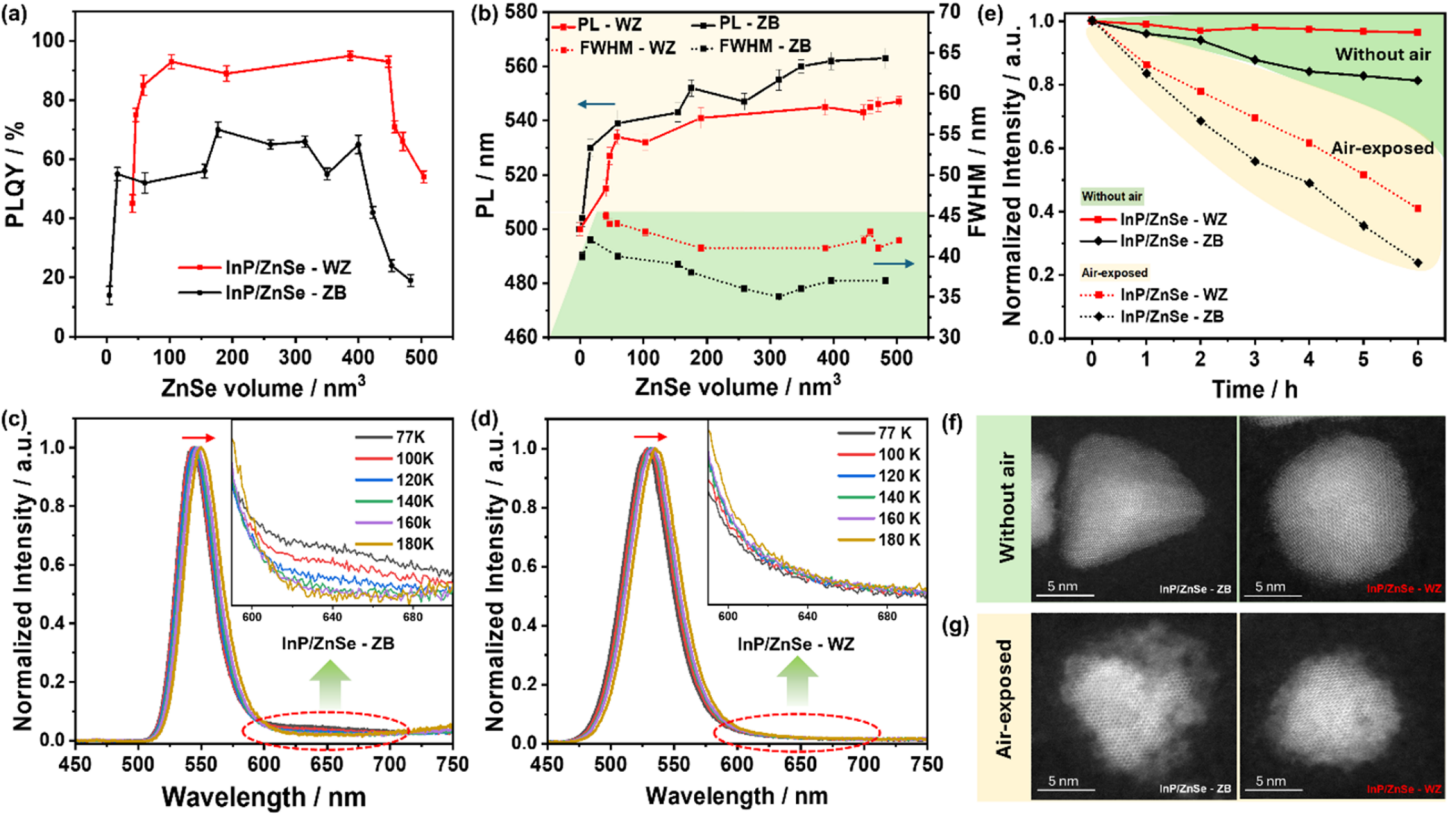
Comparison of optical properties of InP/ZnSe-ZB and InP/ZnSe-WZ QDs. (a) Dependence of PLQY, (b) PL peak, and the full width at half-maximum (FWHM) of InP/ZnSe core/shell QDs on ZnSe shell thickness. The error bars represent the standard deviation across multiple independent syntheses. Temperature-dependent PL emission spectra of (c) InP/ZnSe-ZB and (d) InP/ZnSe-WZ QDs in the temperature range of 77–180 K. (e) Variation of PL intensity of InP/ZnSe-ZB and InP/ZnSe-WZ QDs, before and after being exposed to air, as a function of time. HAADF-STEM images of single InP/ZnSe-ZB and InP/ZnSe-WZ QDs (f) before and (g) after being exposed to air.

Stability of QDs is an important factor that must be considered when utilizing them for various applications. We further investigated the stability of both InP/ZnSe-ZB and InP/ZnSe-WZ QDs. Figure 3e presents the comparative analysis of PL intensity over time under inert conditions and after exposure to air. The PL intensity of InP/ZnSe-WZ QDs, kept without air, decreased by only 3% within 6 h, while that of InP/ZnSe-ZB QDs decreased by 19% under the same conditions. When exposed to air for 6 h, both QD types experienced substantial declines in PL intensity. Specifically, InP/ZnSe-WZ QDs demonstrated a 59% reduction, whereas InP/ZnSe-ZB QDs exhibited a more pronounced decrease of 76%. These findings underscore that, despite both QD types being susceptible to air-induced degradation, InP/ZnSe-WZ QDs manifest enhanced stability relative to InP/ZnSe-ZB QDs, under both inert and oxidative conditions. We note that it is also possible to grow a passivating outer ZnS shell, which can increase their stability.^19,22^ The HAADF-STEM images of single InP/ZnSe-ZB and InP/ZnSe-WZ QDs in Figure 3f,g reveal that both QD types exhibit irregular shapes and rougher surfaces after prolonged exposure to air.^62^ This morphological change is attributed to the formation of surface oxides, which alter the shell’s structure and compromise the ZnSe layer’s protection of the InP core. Considering the positional differences of the InP core within the InP/ZnSe-ZB and InP/ZnSe-WZ structures, it is plausible that these alterations in shell morphology led to varying degrees of protection for the InP core, ultimately resulting in differences in stability.

To further explain the observed differences in the optical properties of the InP/ZnSe-WZ and InP/ZnSe-ZB QDs, we performed effective mass calculations using COMSOL Multiphysics. We simulated the geometry of the InP core, overcoated by a spherical or a tetrahedral ZnSe shell, to simulate the morphologies of the WZ and ZB structures, respectively. Then, we solved the self-consistent Schrödinger–Poisson equations over the mesh points, following our previously reported procedure.^63^ The parameters used for the calculations are detailed in Table S4. From these calculations, we obtained the band gap energies of the QDs. Figure 4a presents the exciton energies of a spherical InP core (diameter of 2.85 nm), coated by a tetrahedral (ZB, in gray) or spherical (WZ, in red) shell of varying thicknesses, where, in the WZ structure, the spherical core is in the center of the sphere and, in the ZB structure, the spherical core is in the centroid of the tetrahedron. In order to account for the different shapes of the ZB and WZ shells, similar volumes of shells were compared. Due to variation in the reported values of the conduction band (CB) offset,^64–67^ a range of values between 0.4 and 0.8 eV were considered (the VB offsets were calculated according to the band gaps and CB offsets).

**Figure 4:**
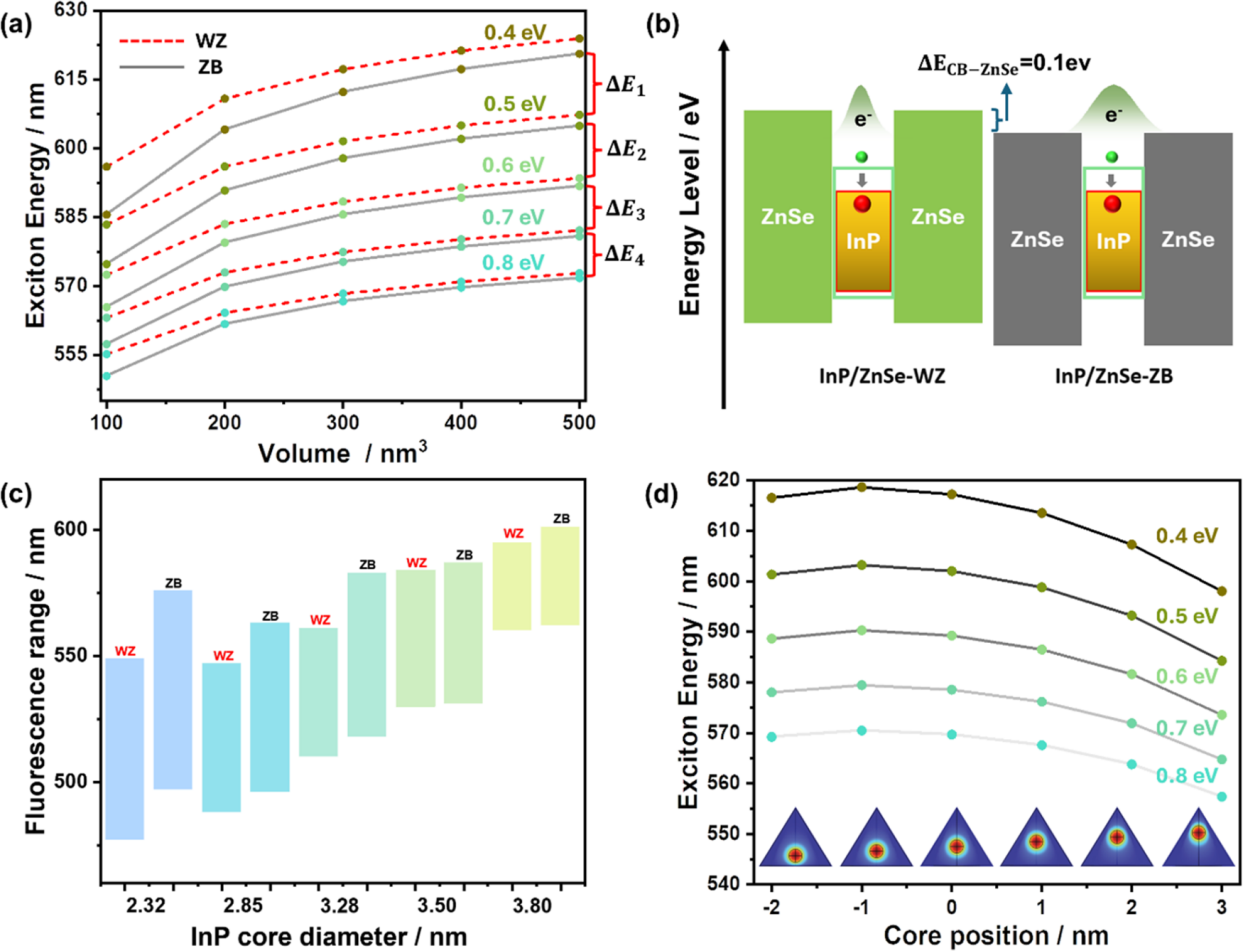
(a) Calculated exciton energies of InP/ZnSe-ZB (gray) and InP/ZnSe-WZ (red) QDs are plotted as a function of the total QDs volume. The simulations consider different conduction band (CB) offsets ranging from 0.4 to 0.8 eV (written on the corresponding curve), corresponding to valence band (VB) offsets from 0.97 to 0.57 eV, in a type-I band alignment. The energy differences Δ*E*_1_ to Δ*E*_4_ represent the exciton energy shifts between InP/ZnSe-ZB and InP/ZnSe-WZ QDs at a ZnSe shell volume of 500 nm^3^ with a conduction band difference of 0.1 eV. (b) Schematic energy level diagrams of InP/ZnSe-ZB and InP/ZnSe-WZ QDs. According to previous reports, the conduction band offset between the two shell phases is approximately 0.1 eV (Δ*E*_CB‑ZnSe_ = 0.1 eV),^66^ indicating slightly weaker electron confinement in the InP/ZnSe-ZB QDs. As the InP core radius decreases, the conduction band edge shifts upward due to quantum confinement, approaching the energy levels of the ZnSe shell. The upward shift may increase the likelihood of electron delocalization into the ZB shell compared to the WZ shell. (c) Evolution range of the fluorescence emission peak during ZnSe shell growth for different InP core sizes. For each core size, the bars represent the emission peak ranges of ZnSe-shelled QDs with either ZB or WZ shell structures. (d) Calculated exciton energies in InP/ZnSe-ZB QDs are plotted as a function of the InP core’s spatial position relative to the centroid of the tetrahedral shell, considering the different band offsets. The core diameter is 2.85 nm, and the total QD volume is fixed at 400 nm^3^. The inset presents slices through the center of the cores showcasing the electron wave functions for each of the calculated core positions (CB offset of 0.4 eV).

The exciton energies that appear in the figure are red-shifted with respect to the experimental band gaps (Figure 3b). This can be attributed to the presence of Zn in the core synthesis, which can result in alloying of the core, which increases the effective band gap.^68^ Nonetheless, for comparing the effects of the different shells, the relative changes provide insights into understanding the differences between the two geometries. Comparing the two geometries, the calculation shows that the WZ structure of a certain volume and band offset is slightly red-shifted relative to the ZB structure. The experimental results, however, exhibit the opposite trend. Nonetheless, the shift between the ZB and WZ structures is relatively small, especially in comparison to the effect of the varying CB offset, which strongly influences the exciton energy. This calls for further examination of the band offset effect as the possible source for variations in the optical properties of ZB- versus WZ-shelled QDs. A study on ZnSe nanowires with alternating WZ and ZB domains revealed a type II band alignment between the two crystal structures.^66^ The ZnSe-ZB structure has lower conduction and valence band energies compared to the WZ structure by up to 98 and 50 meV, respectively.

The studied InP/ZnSe QDs exhibit a type-I band alignment. When considering the decreased CB energy of the ZB-ZnSe, we ultimately obtain a smaller CB offset between the InP core and the ZB-ZnSe shell relative to the larger CB offset between the InP core and the WZ-ZnSe shell (Figure 4b). A smaller CB offset can increase the delocalization of the electron wave function into the shell, thereby reducing the quantum confinement in the bigger “box”. Accordingly, to enable a more accurate comparison between the different geometries, a smaller CB offset for the InP/ZnSe-ZB QDs should be considered. As shown in Figure 4a, a 0.1 eV decrease in the CB offset for the ZB structure is sufficient to obtain a lower exciton energy for this geometry relative to that of the WZ structure. The energy differences Δ*E*_1_ to Δ*E*_4_ represent the exciton energy shifts between InP/ZnSe-ZB and InP/ZnSe-WZ QDs when the ZnSe shell volume is 500 nm^3^, and their CB gap is 0.1 eV (Δ*E*_CB‑ZnSe_). These energy differences gradually decrease as the CB offset increases from 0.4 to 0.8 eV. This trend indicates that as the CB offset increases, the electron wave functions in both structures (ZB- and WZ-shelled QDs) become more confined, leading to a reduced difference in the exciton energy between the two geometries. On the other hand, in the valence band (VB), a decrease in the energy of the ZB-ZnSe will lead to an increase in the VB offset of the InP/ZnSe-ZB QDs, which can enhance the confinement of the hole. However, the energy difference in the valence band is relatively small (up to 50 meV). Moreover, the hole is much heavier than the electron and is thus less sensitive to changes in the potential barrier. Therefore, it is evident that the (conduction) band offset effect is the dominant effect, and the shape effect is much less significant in explaining the optical shifts between the ZB and WZ QDs.

In view of the above discussion, in addition to tuning the shell thickness, it is also straightforward to consider adjusting the band gap of the InP core by changing its size, thereby influencing the energy band alignment between the InP core and the ZnSe shell with a fixed volume. As the core size increases, the band gap of InP narrows, and the localization of the electron wave function within the larger core is enhanced. This effect manifests in the optical properties as a reduced red shift in the emission peak during shell growth. Owing to the small effective mass of electrons in InP QDs (*m*_e_^*^ = 0.073),^12^ the conduction band-edge energy level (1S_e_) is highly sensitive to core size, significantly increasing as the core radius decreases. In contrast, the valence band-edge energy level (1S_h_), associated with a larger hole effective mass (*m*_h_^*^ = 0.45), remains relatively stable. Specifically, according to previous reports,^69^ as the radius of InP QDs increases from 1.2 to 1.9 nm, the 1S_h_ energy changes by less than 0.1 eV, while the 1S_e_ level decreases by approximately 0.5 eV, approaching the conduction band edge of ZnSe (Figure 4b). Therefore, when considering that the CB energy level of the WZ-ZnSe shell is higher than that of the ZB-ZnSe shell, it can be expected that, given the appropriate InP QD band gap, the electrons of the InP core in InP/ZnSe-ZB QDs are more likely to delocalize into the shell region, resulting in a larger red shift in the emission peak compared to InP/ZnSe-WZ QDs (Figure 4b). Furthermore, with the increase in the InP core size and the enhanced electron localization, the spectral shift ranges of InP/ZnSe-ZB and InP/ZnSe-WZ gradually converge.

To examine the proposed effect of core size on the band alignment and electron delocalization between the InP core and ZnSe shell, we synthesized a series of InP cores with varying diameters (Figure S15 shows the absorption spectra in the Supporting Information). The corresponding evolution of the emission peaks for InP/ZnSe-ZB and InP/ZnSe-WZ QDs is summarized in Figure 4c. The experimental results align well with the theoretical understanding based on quantum confinement and band structure considerations: for smaller cores, InP/ZnSe-ZB QDs exhibit a more pronounced red shift than InP/ZnSe-WZ QDs, with the difference gradually diminishing as the core size increases (Figure 4b).

As apparent from the theoretical calculations, in the ZB QDs, deviations of the core position can also highly impact the optical properties. Figure 4d presents the calculated exciton energies of InP cores in tetrahedron shells following changes in the position of the core (core diameter of 2.85 nm and total QD volume of 400 nm^3^). This is another knob for variations in the exciton energy of the ZB QDs, as it appears that the band gap shifts along with the core position. The figure shows that when the core is closer to the tip, the energy is blue-shifted. The electron wave functions in the inset suggest that this is due to a stronger confinement of the wave function when the core approaches the tip of the tetrahedron (Figure S16, Supporting Information). However, as the experimental data suggest, the effect of the core position influences the optical properties in manners that exceed the exciton energetics. The proximity of the core to the surface also influences the exciton dynamics due to interaction with surface defects and traps, which are not accounted for by the simulation. Therefore, the main finding that the simulation reveals is the sensitivity of the exciton energy to the band offset, which is known to differ between the different structures.

To expand the family of such unique core–shell architectures and to demonstrate the generality of this growth behavior in III–V colloidal semiconductor nanocrystals, we employed the same approach for the growth of ZnSe shells on InAs cores, whose emission spectrum can extend into the near-infrared region. The InAs cores were synthesized by the continuous injection process reported previously.^33,34^ The synthesis was stopped when the first exciton peak reached 920 nm, corresponding to a diameter of 3.7 nm (Figures S17a and S18a, Supporting Information). The zinc-blende structure of these InAs cores was confirmed by XRD measurement (Figure S17b, Supporting Information).^70–72^ Similarly, in the absence of TOP during the shell growth process, InAs/ZnSe tetrapod QDs can be successfully synthesized at 300 °C (Figures S18b and S19, Supporting Information), and spherical InAs/ZnSe-WZ core/shell QDs can be obtained by the ripening-assisted process and gradient heating to 340 °C. (Figures 5a,b, and S20a, Supporting Information). The EDS chemical map of InAs/ZnSe-WZ QDs showcases that the InAs core is typically centered within a symmetrically grown ZnSe shell (Figures 5c and S21, Supporting Information). Tetrahedral InAs/ZnSe-ZB QDs can also be synthesized using the aforementioned method (Figures 5d,e and S20b, Supporting Information). As in the case of InP/ZnSe-ZB QDs, it is apparent that the shell grows asymmetrically around the core such that the core is not in the center of the tetrahedron (Figures 5f, and S22, Supporting Information). The XRD measurements in Figure 5g show that the QDs indeed differ in their crystalline structure, where the tetrahedral QDs maintain the zinc-blende phase of the core, and the shell of the spherical QDs has a wurtzite structure.

**Figure 5:**
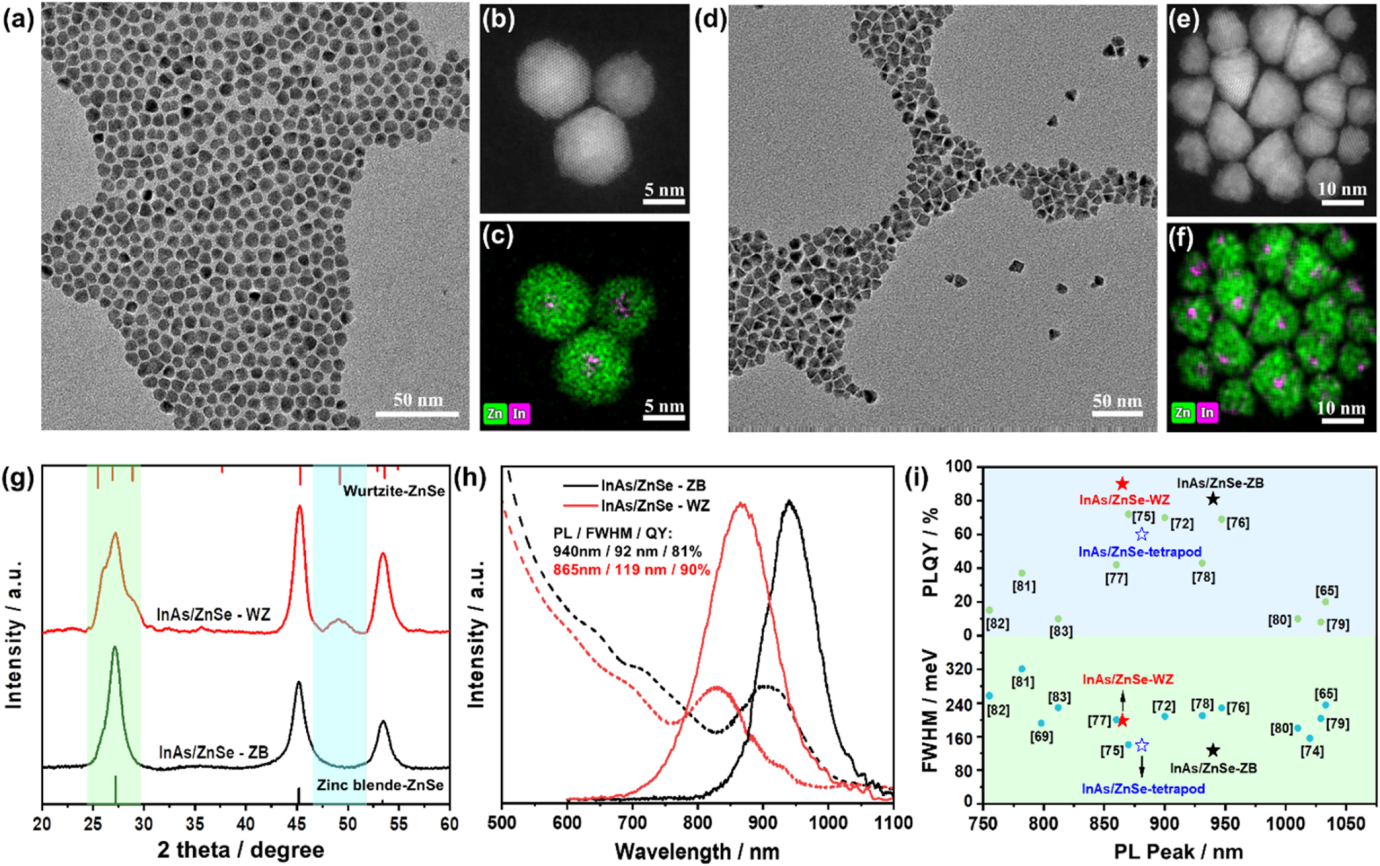
Morphology control of ZnSe epilayers grown on InAs QDs. (a, d) TEM images, (b, e) HAADF-STEM images, (c, f) EDS elemental mapping, (g) XRD patterns, and (h) UV–vis absorption and PL spectra of InAs/ZnSe-ZB and InAs/ZnSe-WZ QDs. (i) PLQY and FWHM of InAs/ZnSe QDs; the reported PLQY and FWHM values for InAs/ZnSe QDs are also displayed for comparison.^65,72–83^

We further compared the optical properties of InAs/ZnSe core/shell QDs prepared using the same amount of the ZnSe shell precursor. As shown in Figure 5h, the fluorescence peak position of InAs/ZnSe-WZ QDs is located at 865 nm, with a FWHM of approximately 119 nm, and the PLQY reaches 90%. The fluorescence peak position of InAs/ZnSe-ZB QDs is positioned at 940 nm, with a FWHM of 92 nm and the PLQY rising to 81%. The time-resolved PL decays of InAs/ZnSe-ZB and InAs/ZnSe-WZ QDs can be well fitted by a monoexponential function (yielding lifetimes of 72 and 68 ns, respectively), indicating pronounced emission of the band-edge excitons (Figure S23, Supporting Information). Notably, these QDs present high PLQY and narrow FWHM in the near-infrared region, which can be advantageous compared with other InAs/ZnSe QDs reported in the literature recently (Figure 5i, Table S5, Supporting Information).^65,72–83^

## Conclusions

In summary, we developed the morphology and crystal phase control methods of the ZnSe epitaxial layers grown on III–V QDs. The presence of TOP lowers the Se precursor reactivity and, through its coordination behavior, enables switchable control between thermodynamic and kinetic growth regimes. Under low activity, growth favors thermodynamically stable [111] facets, forming symmetric tetrahedral nanocrystals. At high activity, kinetic control dominates, leading to anisotropic tetrapod-like structures. Combined with the assistance of Ostwald ripening, while the shell morphology changes from tetrapod to spherical, its shell crystal structure also changes from zinc-blende to wurtzite. A comparison of the optical properties revealed that spherical-WZ structures offer superior passivation and protection of the core, markedly enhancing PLQY and stability. Quantum mechanical simulations indicate that variations in the band offsets between the ZB and WZ structures can tune the band gaps of the QDs, resulting in shifted exciton energies. Interestingly, the core position within the core–shell QDs under different growth modes further affects the tunability of its emission spectrum and exciton dynamics. Combining experiments and modeling, this work enhances and expands the control of photophysical properties of III–V core/shell structure QDs. It provides a promising general strategy for obtaining high-quality III–V core/shell QDs with excellent performance, offering significant potential for optoelectronic and bioimaging applications.

## Materials and Methods

### Materials

Indium­(III) acetate (In­(Ac)_3_, 99.99% trace metals basis), palmitic acid (PA, 99%), oleic acid (OA, 90%), zinc acetate (Zn­(Ac)_2_, 99.99%), 1-octadecene (90%, ODE), trioctylphosphine (97%, TOP), tris­(trimethylsilyl)-phosphine (98%, (TMS)_3_P), tris­(trimethylsylil)­arsine (99%, (TMS)_3_As), zinc stearate (Zn­(St)_2_, 10–12% Zn basis), squalane (90%), selenium (powder, 100 mesh, 99.99% trace metals basis), toluene (anhydrous, 99.8%), and ethanol (anhydrous, 99.8%) were purchased from Sigma-Aldrich. All precursors and solvents were used without further purification. All solvents were stored inside an N_2_-filled glovebox.

### Instrumentation

The measurements of UV–vis absorption were performed on a Jasco V-570 UV–vis-NIR spectrophotometer. Fluorescence spectra were measured with a Varian Cary Eclipse spectrophotometer. Time-resolved PL measurements were carried out on an Edinburgh FLS920 fluorescence spectrometer. Photoluminescence quantum yield was measured with a Hamamatsu Absolute PL quantum yield spectrometer C11347–11. Powder X-ray diffraction (XRD) patterns were recorded on a Philips PW1820 diffractometer using Cu Kα photons. Transmission electron microscopy (TEM) images were performed using a Tecnai G2 Spirit Twin T-12 microscope (Thermo Fisher Scientific) operated at 120 kV. High-resolution high-angle annular dark-field (HAADF) scanning TEM (STEM) images and elemental mapping were obtained on Themis Z G3 (Thermo Fisher Scientific) at 300 keV. The images and EDS maps were obtained and analyzed with Velox software (Thermo Fisher Scientific). The temperature-dependent PL spectra were measured by a HORIBA JOBIN YVON Fluoromax-4 spectrofluorometer at different temperatures in a liquid nitrogen cryostat.

### Synthesis of InP Core QDs

InP QDs were prepared following the previously reported method with minor modifications.^31,32^ For a typical synthesis, 0.4 mmol of In­(Ac)_3_, 0.1 mmol of Zn­(Ac)_2_, 1.4 mmol of PA, and 10 mL of ODE were mixed in a 50 mL flask. The mixture was heated to 120 °C under vacuum for 1 h and then refilled with argon and cooled to 50 °C. Subsequently, 0.32 mmol of (TMS)_3_P mixed with 2 mL of TOP was injected quickly into the flask. After injection, the temperature was raised to 270 °C and maintained for 3 min. When the desired size was reached, the reaction was quenched by cooling the solution to room temperature. To prepare InP QDs of different sizes, InP clusters were first synthesized using the same precursors and ratios as described above, except that the temperature was maintained at 30 °C during (TMS)_3_P injection. After stirring for 1 h, the solution was transferred to a glovebox for storage. Subsequently, the InP seeds prepared by this method were grown by dropwise addition of the precursor at 300 °C at a rate of 2 mL/h while monitoring the reaction progress via absorption spectroscopy. When the first exciton peak reached the desired position, the heating mantle was removed, and the reaction was terminated by decreasing the temperature. To purify the InP core QDs, 10 mL of the crude solution was mixed with 10 mL of toluene in a 50 mL centrifuge tube. Then, 20 mL of ethanol as an antisolvent was added to precipitate the InP core QDs, followed by centrifugation at 6000 rpm for 5 min. The supernatant was discarded, and the sediment of InP core QDs was redispersed in 5 mL of toluene. The washing process was repeated 2 times. Finally, the purified InP core QDs were dissolved in toluene and stored in a glovebox for the shell growth step. The washing process was performed in a glovebox filled with nitrogen or sealed vials.

### Synthesis of InAs Core QDs

InAs QDs were synthesized according to a literature method.^30,31^ Both InAs clusters and InAs QDs were prepared by using the same precursor. For the InAs clusters solution, 12 mmol In­(Ac)_3_ and 36 mmol OA were mixed with 60 mL of ODE. The mixture was degassed at 120 °C under vacuum for 90 min and then cooled down to room temperature under Argon. 1.6 g of (TMS)_3_As was mixed with 12 mL of dry ODE in the glovebox. The As solution was injected at room temperature into the indium-oleate solution with constant stirring. The temperature slowly rose to 80 °C until the solution turned dark red. The InAs nanoclusters were cooled to room temperature and kept under an inert atmosphere in the glovebox. For the InAs QDs, 2 mmol of In­(Ac)_3_ and 6 mmol of OA were mixed with 5 mL of ODE and left under vacuum at 120 °C for 2 h, and then switched to Argon, and the temperature was raised to 300 °C. In the glovebox, 0.24 g of (TMS)_3_As was mixed with 2 mL of dry ODE. The As solution was injected at 300 °C into the indium-oleate solution with constant stirring to form InAs seeds. The temperature was lowered to 285 °C and maintained constant for about 20 min. During this time, aliquots were taken, and their absorption spectrum was measured. Once the first exciton absorption feature of the seeds reached ≈750 nm, the nanoclusters solution was added continuously at a constant rate of 2 mL·h^–1^. During this reaction, aliquots were taken at constant intervals to evaluate the growth and size focusing of the nanocrystals by absorption measurements. At the end of the synthesis, the cooled solution was transferred into the glovebox, and successive precipitation–centrifugation–redispersion cycles were performed in order to clean the nanocrystals from the crude solution using dry toluene and ethanol as the solvent and antisolvent, respectively. The clean nanocrystals were dispersed in toluene and kept in a glovebox for further use and analysis.

### Synthesis of Tetrahedral InP/ZnSe or InAs/ZnSe QDs

A 0.2 mmol amount of Zn­(St)_2_, 1 mL of TOP, 2 mL of squalane, and the purified InP or InAs core QDs in toluene were loaded into a 50 mL three-neck flask and degassed at 120 °C for 1 h under vacuum. After backfilling with argon, the reaction solution was heated up to 300 °C. The Se suspension (Se-SUS, 0.2 mL, 0.5 M) was added to the solution and held at that temperature for 30 min. When the temperature of the solution in the flask reached 340 °C, 0.4 mL of a 0.4 M Zn­(St)_2_ suspension and 0.2 mL of a 0.5 M Se-SUS solution were separately injected dropwise into the flask every 30 min. Aliquots were taken to monitor the shell growth progress. The obtained InP/ZnSe or InAs/ZnSe QDs were purified with toluene and ethanol and dispersed in toluene. All of the washing procedures were performed in the glovebox.

### Synthesis of Spherical InP/ZnSe or InAs/ZnSe QDs

For the synthesis of InP/ZnSe or InAs/ZnSe spherical QDs, 0.2 mmol of Zn­(St)_2_, 3 mL of squalane, and the purified InP or InAs core QDs in toluene were loaded into a 50 mL three-neck flask and degassed at 120 °C for 1 h under vacuum. After backfilling with argon, the reaction solution was heated up to 300 °C. The Se-SUS (0.2 mL, 0.5 M) was added into the solution and held at that temperature for 30 min. The temperature increased to 340 °C under the argon, and it was maintained for 30 min. Then, 0.4 mL of a 0.4 M Zn­(St)_2_ suspension and 0.5 mL of a 0.2 M Se-SUS solution were separately injected dropwise into the flask every 30 min. Aliquots were taken to monitor the shell growth progress. The obtained InP/ZnSe or InAs/ZnSe QDs were purified with toluene and ethanol and dispersed in toluene. All of the washing procedures were performed in the glovebox.

## Supplementary Material



## Notes

Characteristics of InP QDs; STEM-EDS elemental mapping and HRTEM images of the InP/ZnSe-WZ and InP/ZnSe-ZB QDs; characteristics of InP/ZnSe core–shell QDs synthesized at different reaction temperatures; characteristics of InP/ZnSe QDs as the annealing time increases; material parameters used in simulations; and characterization, TEM images, XRD patterns, absorption and PL spectra of InAs/ZnSe QDs (PDF)
