# Supplementary material for: Shell Phase and Morphology Control for Emission Tuning in III–V Core/Shell Quantum Dots
Source: ACS Nano. 2025 Aug 5;19(32):29765–77. doi: 10.1021/acsnano.5c10168 (PMC12368997; doi:10.1021/acsnano.5c10168)
Supplement: Supplementary file 1 [file nn5c10168_si_001.pdf]

Supporting Information

Shell Phase and Morphology Control for Emission Tuning in III-V Core/Shell Quantum Dots

Xiang Li, Einav Scharf, Adar Levi, Yinon Deree, David Stone, Sergei Remennik, Uri Banin*

The Institute of Chemistry and The Center for Nanoscience and Nanotechnology

The Hebrew University of Jerusalem

Jerusalem 91904, Israel

Corresponding Author

*E-mail: uri.banin@mail.huji.ac.il

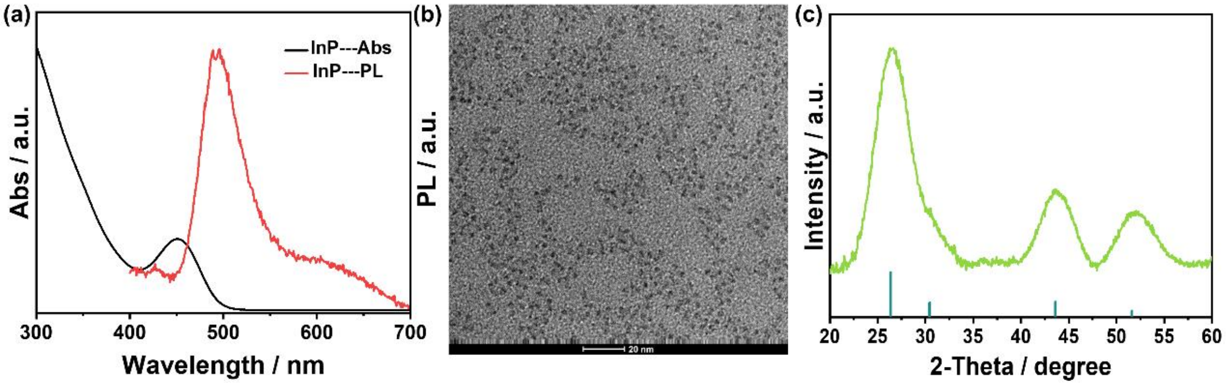

Figure S1. Characteristics of InP QDs. (a) Absorption and PL spectra, (b) TEM image and (c) XRD patterns of InP core. The vertcal bars indicate the characteristic peaks of bulk zinc blende InP (JCPDS No. 32-0452).

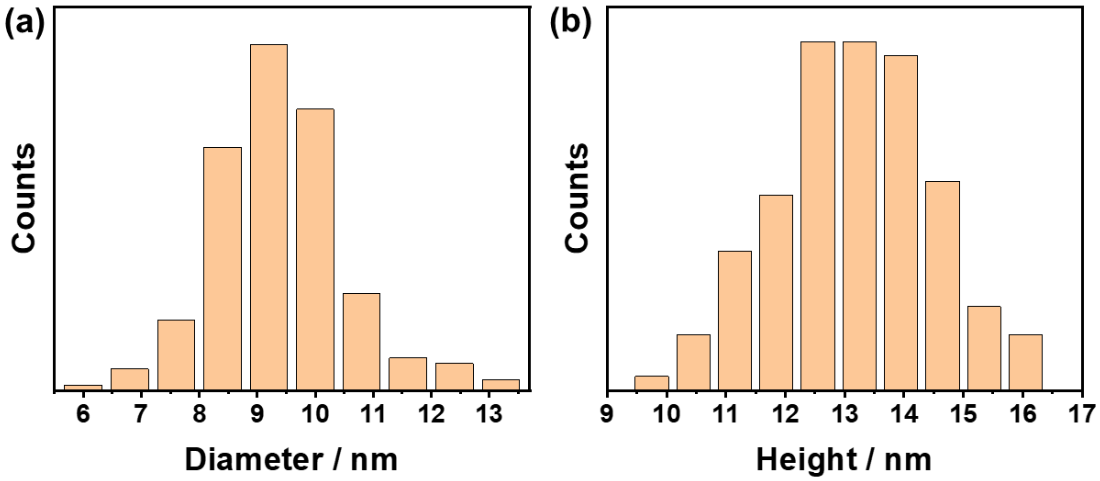

Figure S2. Size distribution histograms and TEM images of the (a)InP/ZnSe -WZ and (b)InP/ZnSe -ZB QDs from Figure 1h and 1j in the main text.

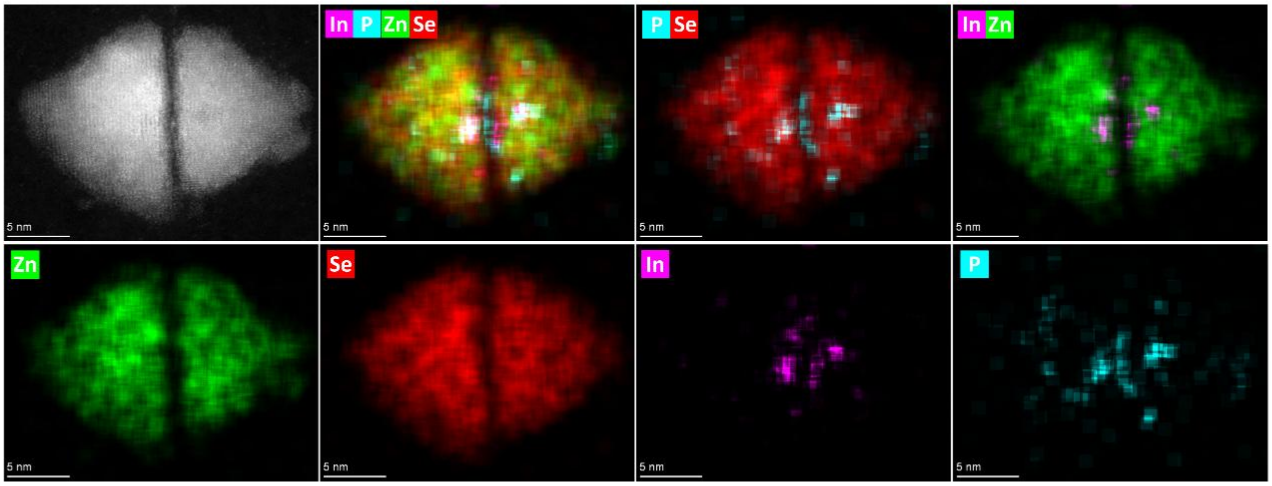

Figure S3. STEM-EDS elemental mapping of the InP/ZnSe-ZB QDs.

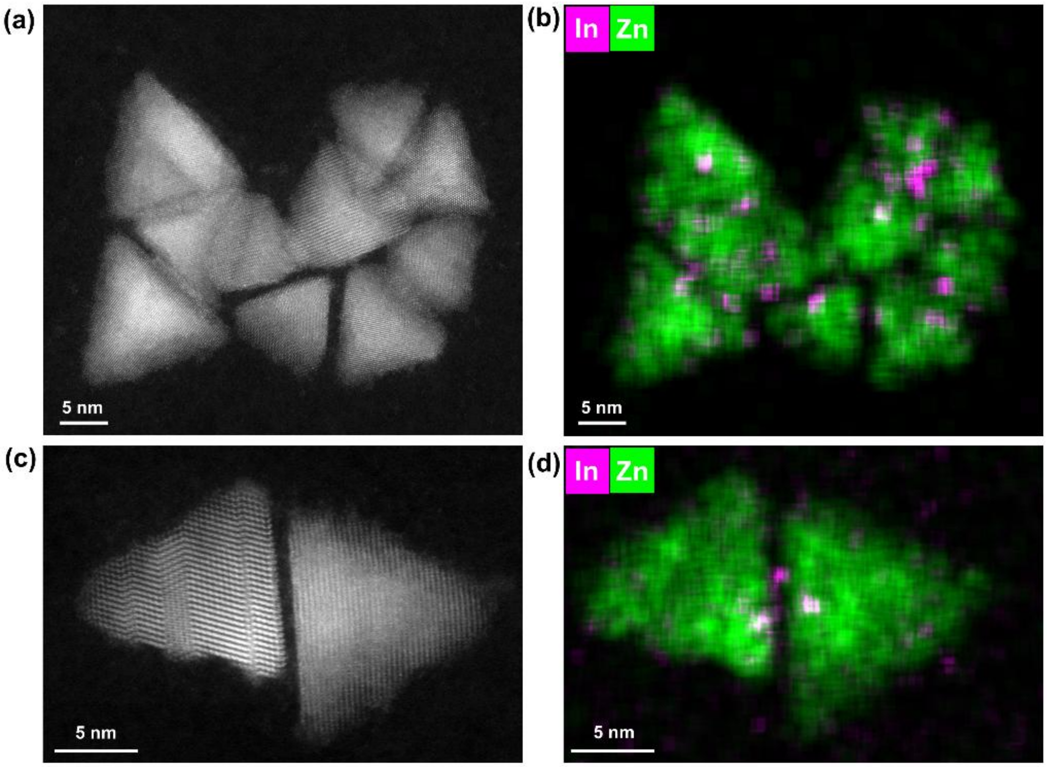

Figure S4. (a,c) HAADF-STEM image and corresponding (b,d) EDS elemental mapping of InP/ZnSe-ZB QDs.

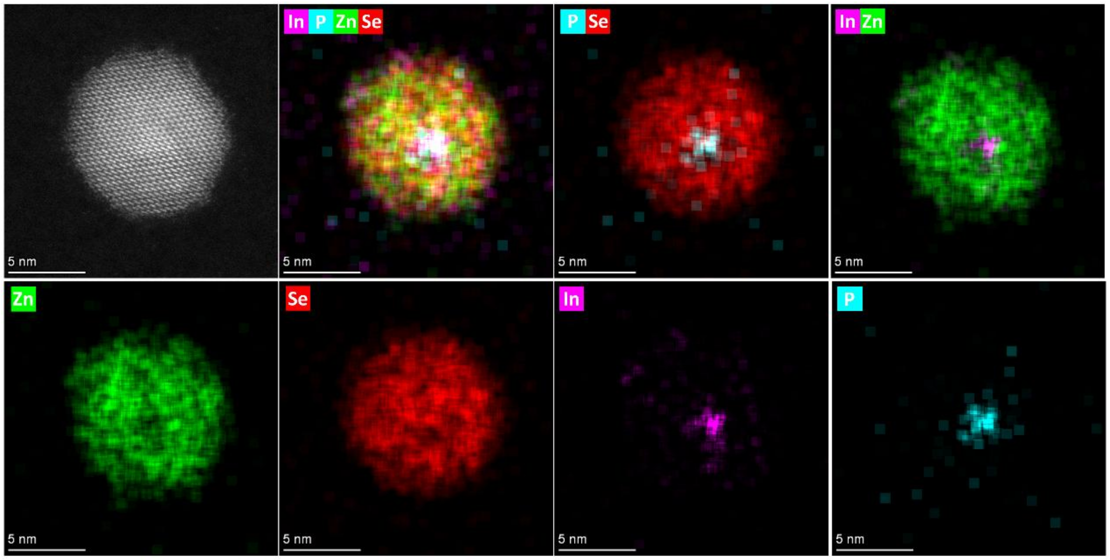

Figure S5. STEM-EDS elemental mapping of the InP/ZnSe-WZ QDs.

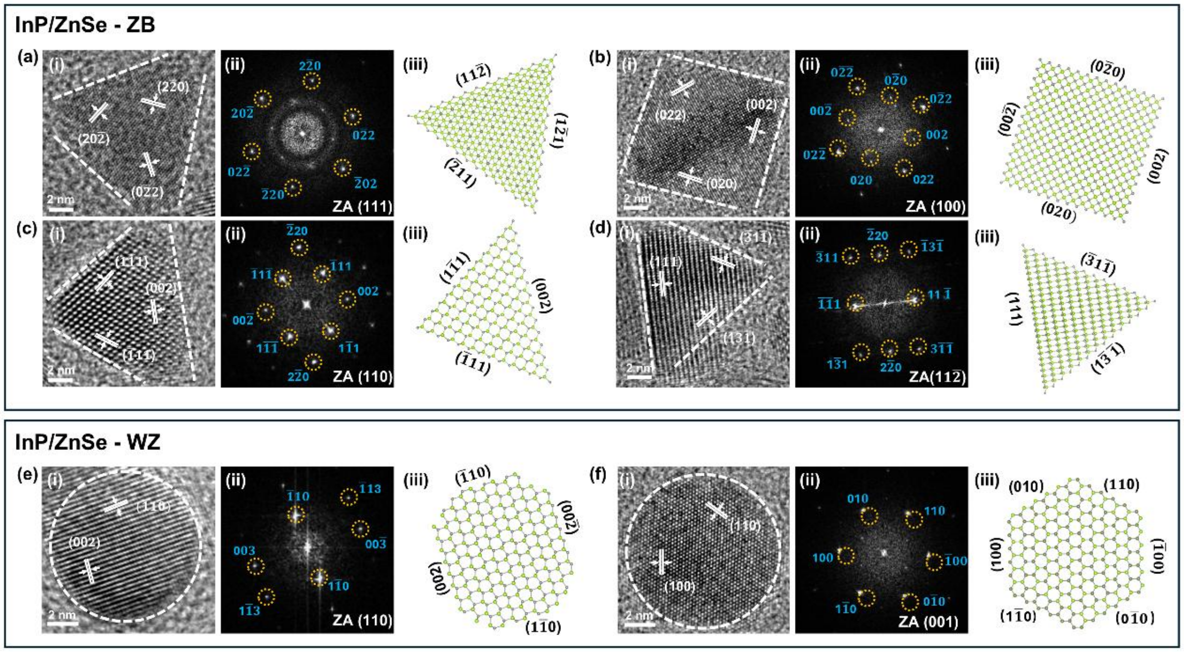

Figure S6. (i) The HR-TEM images, (ii) related FFT pattern and (iii) structure model of tetrahedral (a-d) InP/ZnSe-ZB QDs viewed under ZA of [111], [100], [110], and [11$\bar{2}$], respectively. And the HR-TEM images (i) and related FFT pattern (ii) of spherical (e-f) InP/ZnSe-WZ QDs viewed under ZA of [110] and [001], respectively.

***Calculation of the Volume of Tetrahedral InP/ZnSe-ZB**.

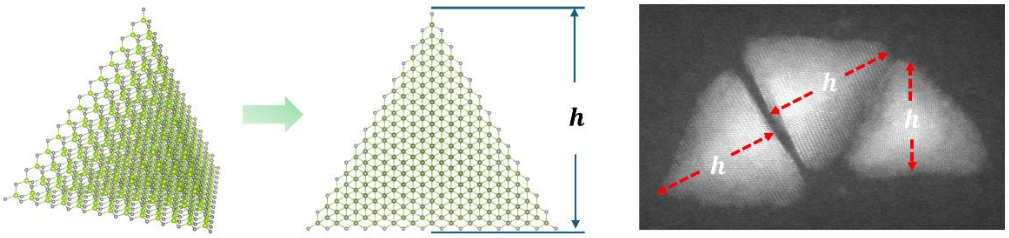

Experimentally, we measured the height h of the tetrahedron nanocrystals from TEM images and calculated their total volume using the following formula: $V = \frac{2\sqrt{6}}{27}h^3$

The InP core is assumed to be quasi-spherical, with a volume given by: $V_{core} = \frac{4}{3}\pi r^3$, where r is the radius of the InP core. Accordingly, the volume of the ZnSe shell is determined by subtracting the core volume from the total volume: $V_{ZnSe} = V - V_{core} = \frac{2\sqrt{6}}{27}h^3 - \frac{4}{3}\pi r^3$

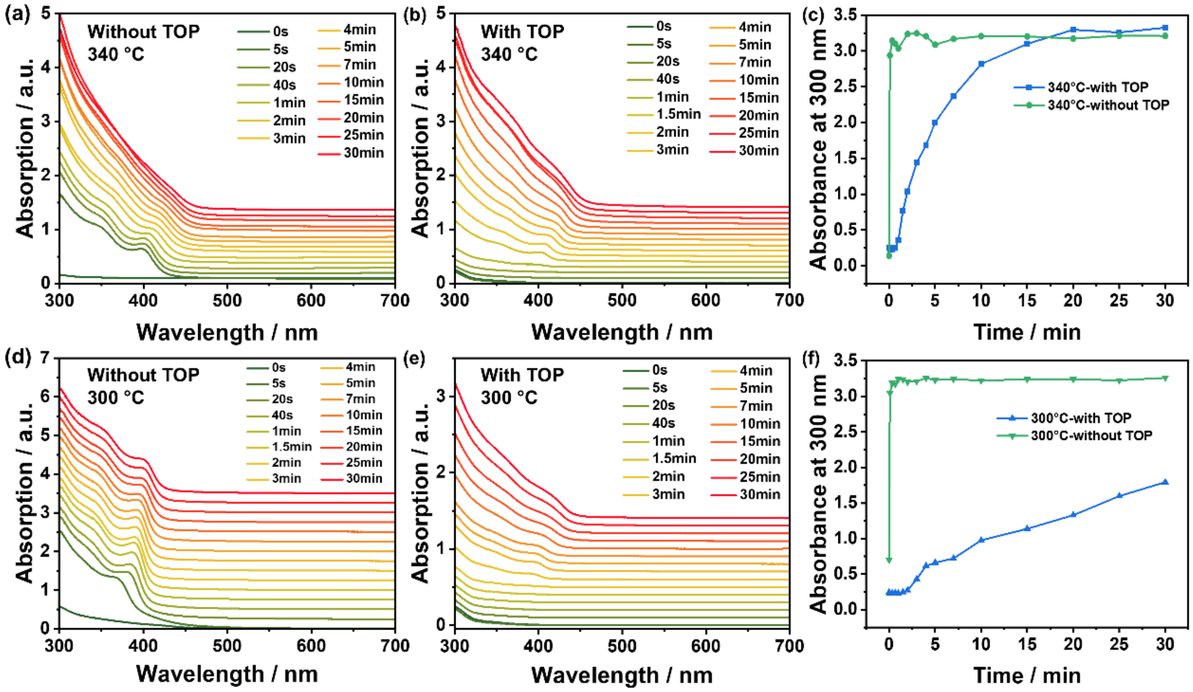

Figure S7. The characterization of ZnSe nucleation obtained (a, d) without or (b, e) with TOP in a one-step hot injection synthesis. The UV-vis absorption spectra of aliquots taken at indicated time from the reaction between $[Zn(St)_2]$-ODE complex and Se-SUS at 340 ºC and 300 ºC, respectively. (c, f) Absorbance at 300 nm of the reaction solution versus reaction time for the synthesis of ZnSe nanocrystals.

Table S1. PL decay parameters, QY, and the calculated radiative and non-radiative recombination rate constants of InP/ZnSe-WZ and InP/ZnSe-ZB QDs. The ZnSe shell volume of both samples is approximately 400nm^3. The corresponding PL decay curves are presented in figure 1n of the main text.

**Samples**	**τ_1 (a_1)**	**τ_2 (a_2)**	**τ_{ave} / ns**	**QY / %**	**k_{rad} / ns^{-1}**	**k_{nrad} / ns^{-1}**
**InP/ZnSe-WZ**	**43 (65%)**	**120 (35%)**	**89**	**92**	**1.03×10^{-2}**	**9.4×10^{-4}**
**InP/ZnSe-ZB**	**33 (45%)**	**116 (55%)**	**100**	**47**	**4.7×10^{-3}**	**5.3×10^{-3}**

τ_i values are represented in ns scale, a_i represents the weightage of a particular lifetime component. QY is PL quantum yield. The PL decay curves were fitted with a bi-exponential function:

$$R(t) = a_1 \exp\left(-\frac{t}{\tau_1}\right) + a_2 \exp\left(-\frac{t}{\tau_2}\right)$$

The corrected lifetime components and amplitudes were used for calculating the fluorescence lifetimes. The average lifetime (τ_{ave}) can be calculated according to the equation:

$$\tau_{ave} = \left(\frac{a_1\tau_1^2 + a_2\tau_2^2}{a_1\tau_1 + a_2\tau_2}\right)$$

The radiative (k_{rad}) and non-radiative (k_{nrad}) recombination rate constants were derived from the average lifetime and QY using the following relationships:

$$k_{rad} = \frac{QY}{\tau_{ave}}\ ,\ k_{nrad} = \frac{1-QY}{\tau_{ave}}$$

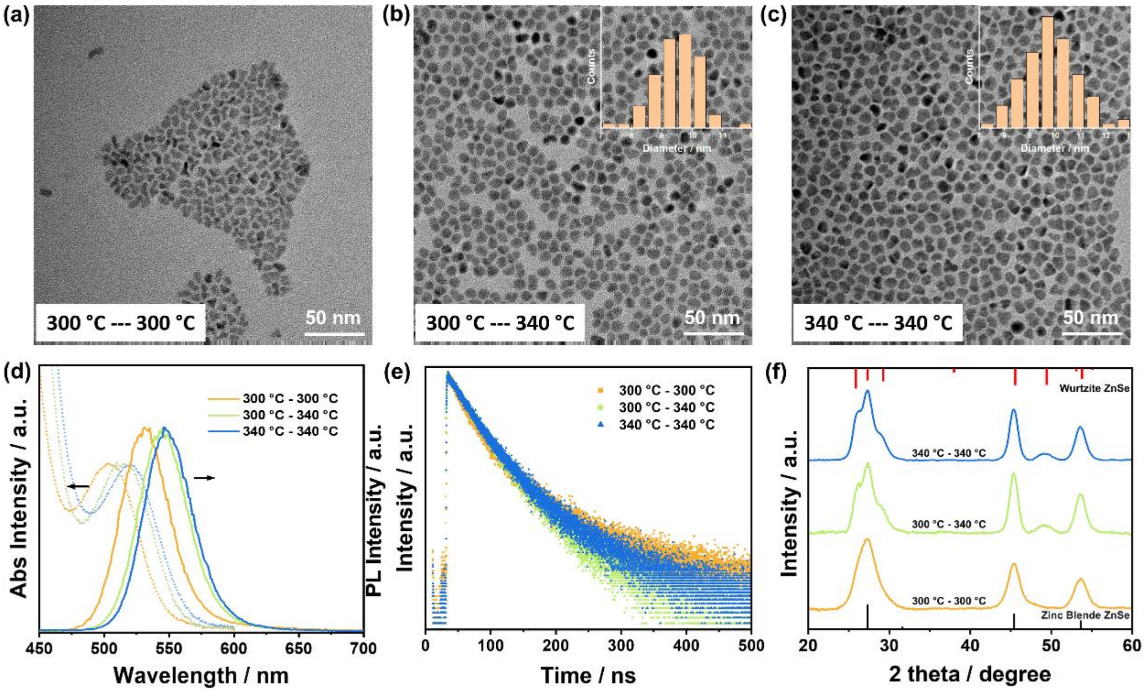

Figure S8. (a-c) TEM images, (d) UV-Vis absorption (dash line) and PL spectra (solid line), (e) PL decay traces, (f) XRD patterns of InP/ZnSe core-shell QDs synthesized at different reaction temperatures.

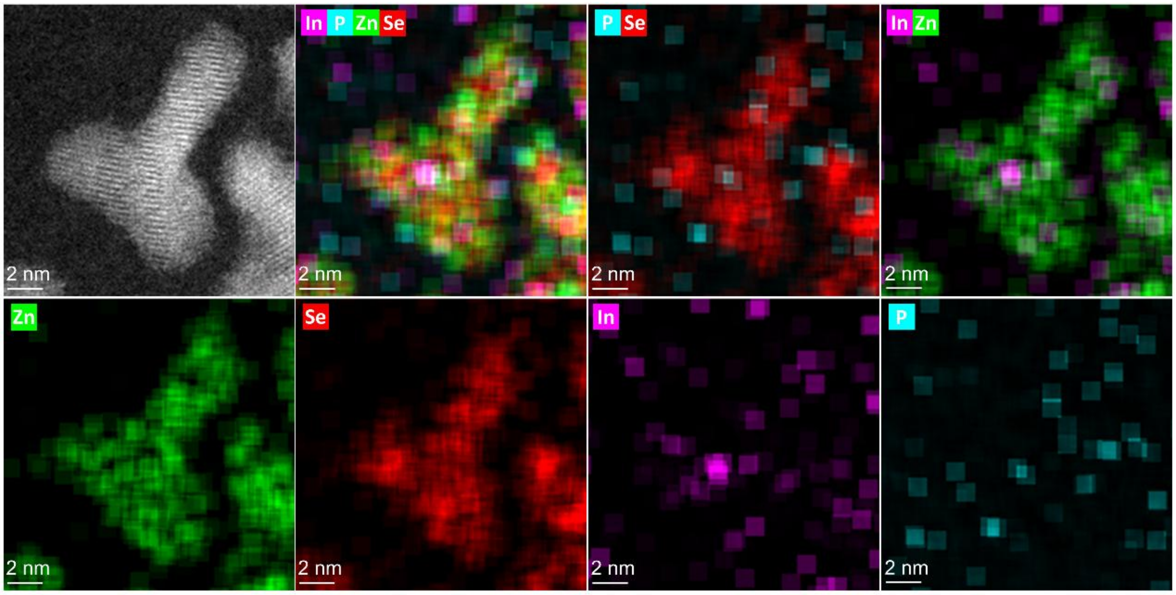

Figure S9. STEM-EDS elemental mapping of the InP/ZnSe - tetrapod QDs.

Table S2. Photoluminescence decay and spectral parameters of InP/ZnSe QDs obtained at different synthesis temperatures. The InP/ZnSe QDs prepared here did not undergo a ripening operation and were intended for comparison of morphology and optical properties under different temperature conditions.

Temperature	τ_1 (a_1)	τ_2 (a_2)	τ_{ave} / ns	PL / nm	FWHM / nm	QY / %
300°C-300°C	33(45%)	110(55%)	95	531	44	71
300°C-340°C	43(60%)	105(40%)	81	545	43	85
340°C-340°C	43(58%)	118(42%)	93	546	45	80

τ_i values are represented in ns scale, a_i represents the weightage of a particular lifetime component. PL, photoluminescence. QY, quantum yield. The corrected lifetime components and amplitudes were used for calculating the fluorescence lifetimes. The average lifetime of the bi-exponential function can be computed as:

$$\tau_{ave} = (\frac{a_1\tau_1^2 + a_2\tau_2^2}{a_1\tau_1 + a_2\tau_2})$$

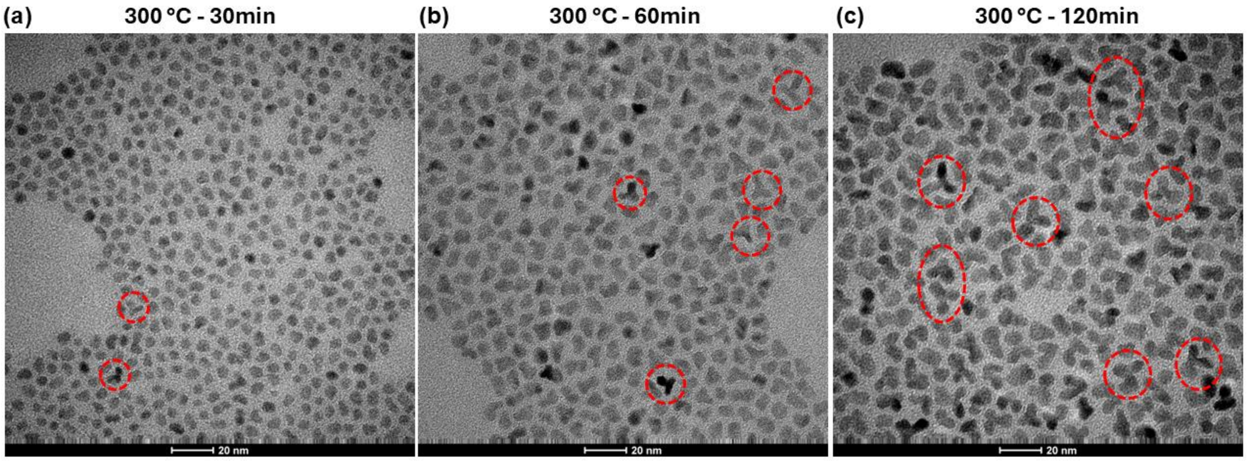

Figure S10. Morphological evolution of the ZnSe shell in InP/ZnSe quantum dots at 300 °C: (a) 30 min, (b) 60 min, and (c) 120 min.

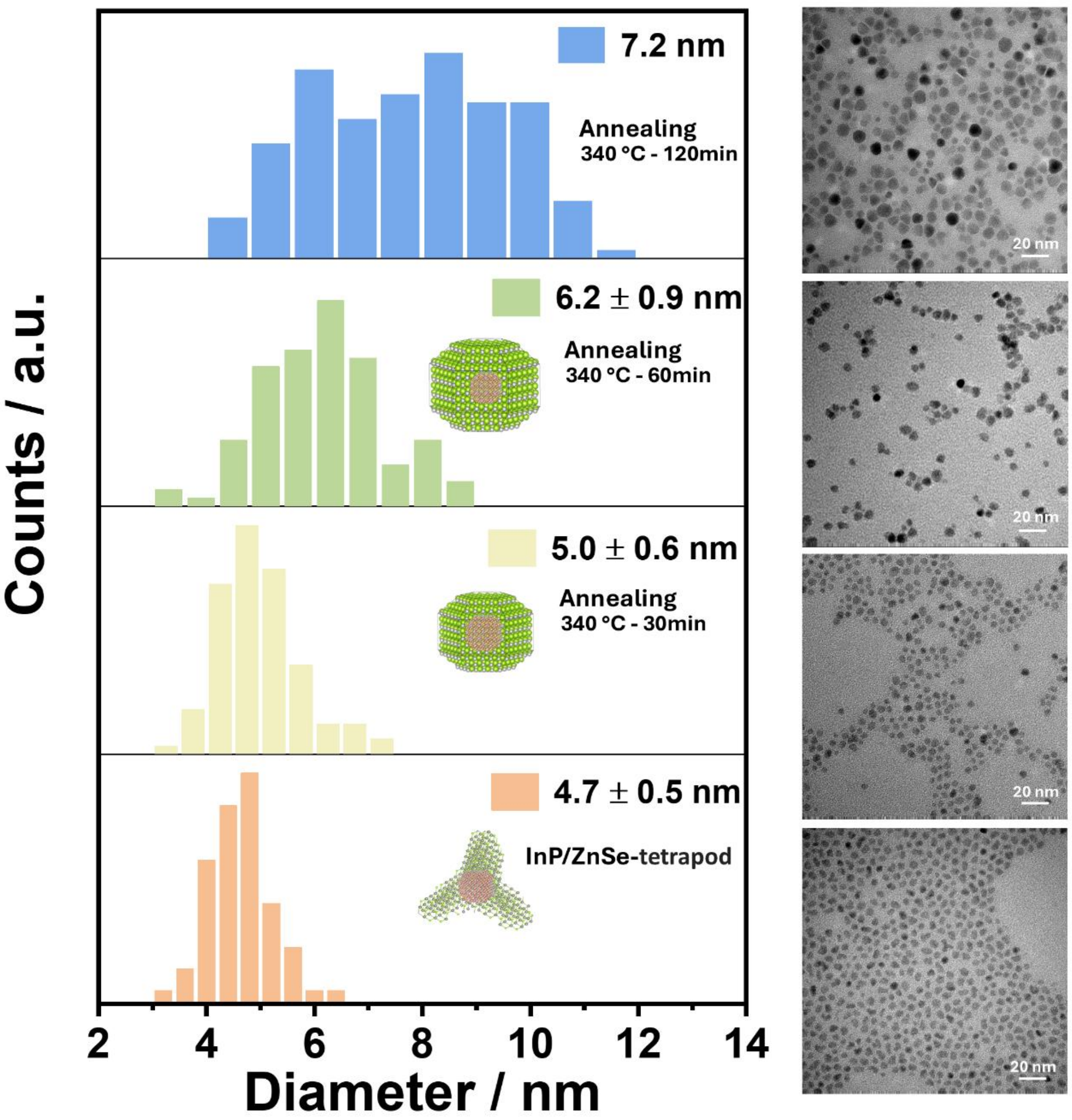

Figure S11. Size distribution histograms and TEM images of the InP/ZnSe QDs as the annealing time increases.

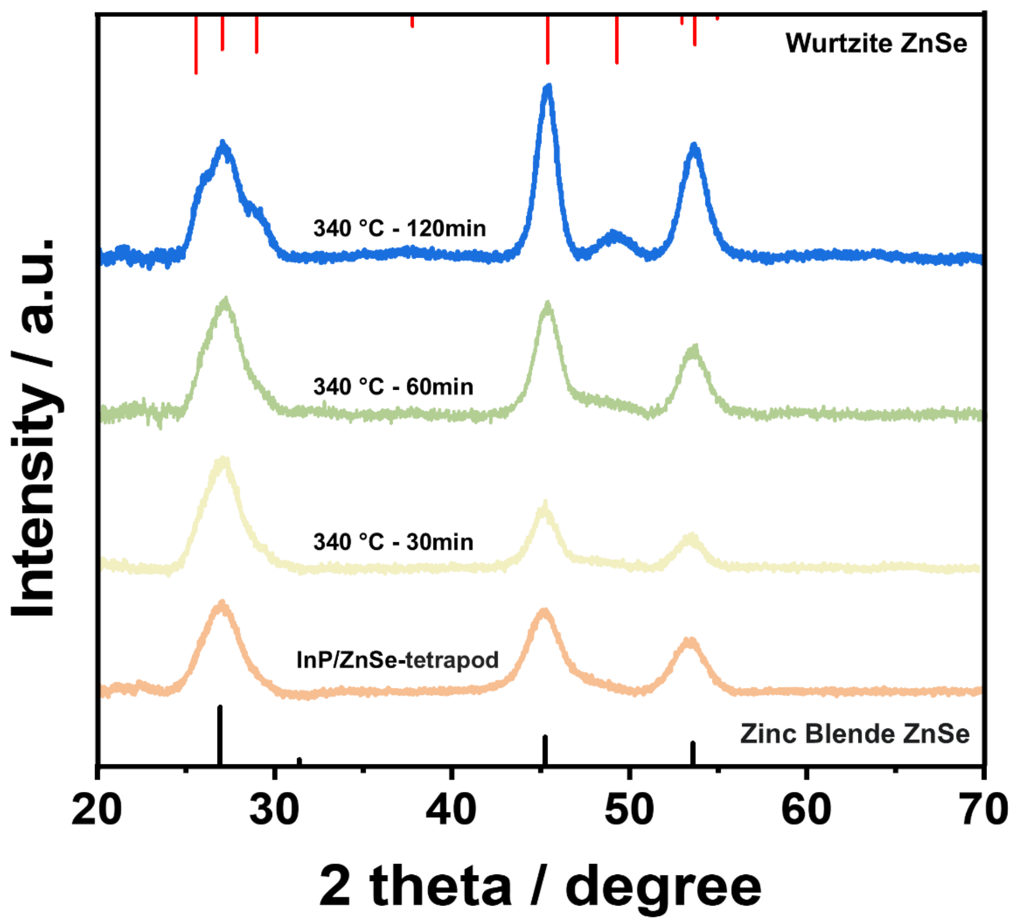

Figure S12. XRD patterns of the InP/ZnSe QDs as the annealing time increases.

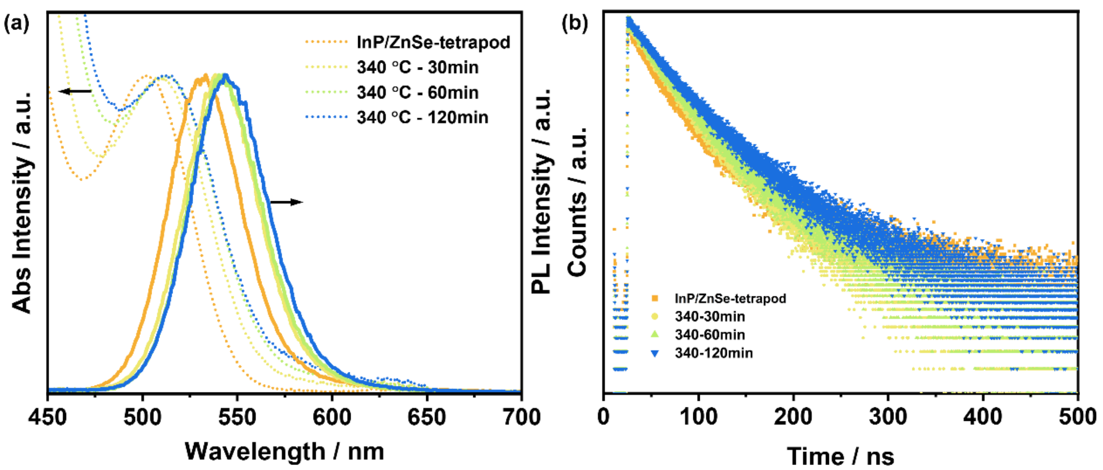

Figure S13. (a) UV–Vis absorption and PL spectra, (b) PL decay traces of the InP/ZnSe-tetrapod QDs as the annealing time increases.

Table S3. Photoluminescence decay and spectral parameters of InP/ZnSe-tetrapod as the annealing time increases.

Samples	τ_1 (a_1)	τ_2 (a_2)	τ_{ave} / ns	PL / nm	FWHM / nm	QY / %
InP/ZnSe-tetrapod	32 (55%)	103 (45%)	83	532	45	65
340°C-30min	37 (64%)	92 (36%)	69	538	46	82
340°C-60min	37 (58%)	94 (42%)	74	542	46	76
340°C-120min	42 (58%)	110 (42%)	87	543	49	69

τ_i values are represented in ns scale, a_i represents the weightage of a particular lifetime component. PL, photoluminescence. QY, quantum yield. The corrected lifetime components and amplitudes were used for calculating the fluorescence lifetimes. The average lifetime of the bi-exponential function can be computed as:

$$\tau_{ave} = \left(\frac{a_1\tau_1^2 + a_2\tau_2^2}{a_1\tau_1 + a_2\tau_2}\right)$$

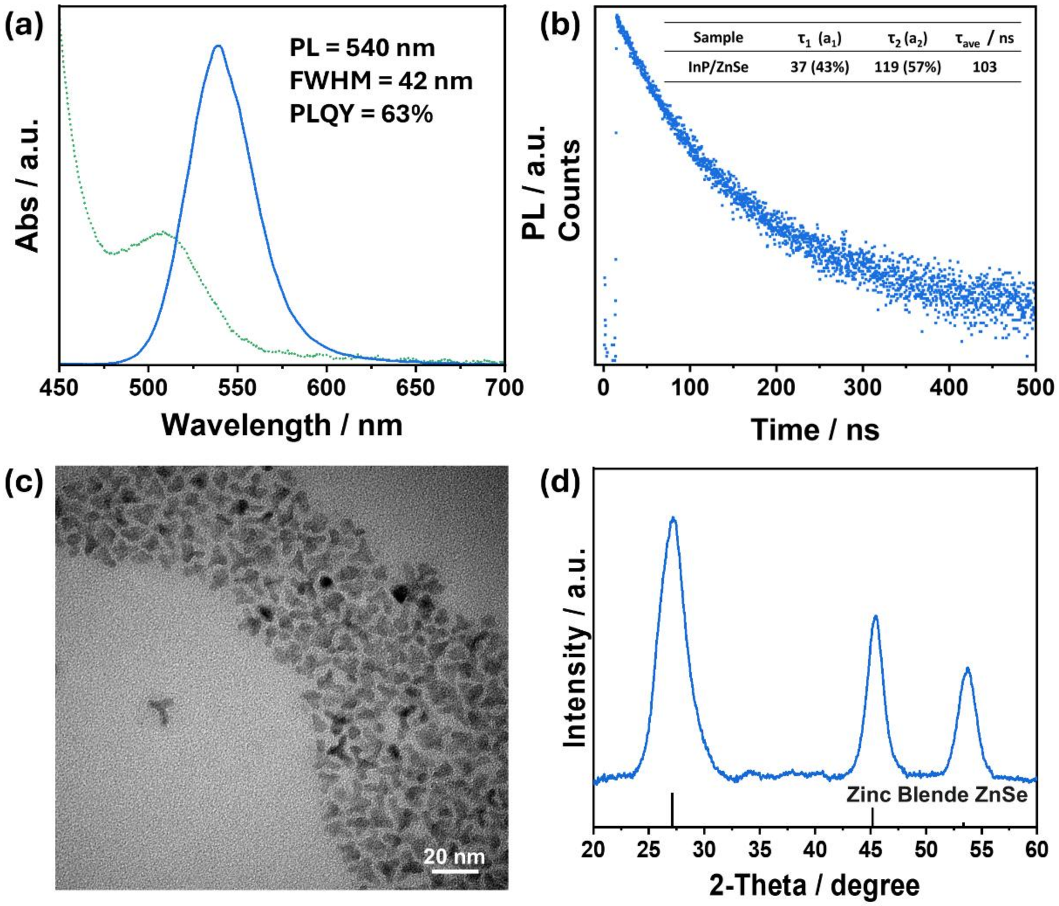

Figure S14. (a) UV-Vis absorption (dash line) and PL spectra (solid line), (b) PL decay traces, (c) TEM images, and (d) XRD patterns of InP/ZnSe core-shell QDs. The InP/ZnSe core-shell QDs were initially annealed at 340 °C for 30 minutes, followed by subsequent growth at 300 °C.

Table S4. Material parameters used in simulations.

Parameter	Value	Units	Reference
**InP E_g**	**1.34**	**[eV]**	**1**
**ZnSe E_g**	**2.71**	**[eV]**	**1**
**InP m_e^***	**0.073**	m_0	**2**
**InP m_h^***	**0.45**	m_0	**2**
**ZnSe m_e^***	**0.16**	m_0	**2**
**ZnSe m_h^***	**0.75**	m_0	**2**
**InP dielectric constant**	**12.56**	**-**	**2**
**ZnSe dielectric constant**	**8.6**	**-**	**2**
**Conduction band offset 1**	**0.41**	**[eV]**	**3**
**Conduction band offset 2**	**0.75**	**[eV]**	**4**

The calculations of the exciton energies and charge carriers' wave functions are simulated by COMSOL Multiphysics, following our previously reported procedure. [5]

According to the previously reported conduction band offsets in table S4, we used values of 0.4, 0.5, 0.6, 0.7, and 0.8 eV in our calculations. The valence band offsets were calculated as the difference between the band gap of the shell and the summation of the bandgap of the core and the conduction bands offset, resulting in 0.97, 0.87, 0.77, 0.67, and 0.57 eV, respectively. The potential barrier between the shell and the environment was set to 2.5 eV. In all calculations, the core is a sphere with a diameter of 2.85 nm. In Figure 4c in the main text, the edge of the tetrahedron is 15.03 nm and the volume is 400 nm^3.

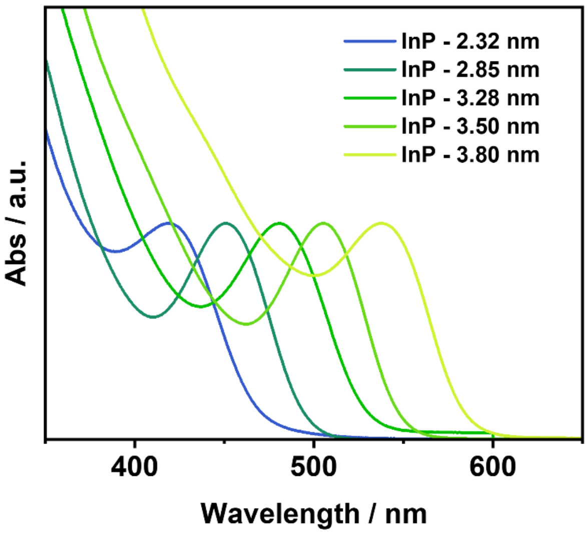

Figure S15. UV–Vis absorption of InP QDs with different sizes, the size of InP QDs is calculated by the first exciton absorption peak and the following equation:

$D = (-3.7707 \times 10^{-12})\lambda^5 + (1.0262 \times 10^{-8})\lambda^4 - (1.0781 \times 10^{-5})\lambda^3 + (5.4550 \times 10^{-3})\lambda^2 - (1.3122)\lambda + 119.9$ [6]

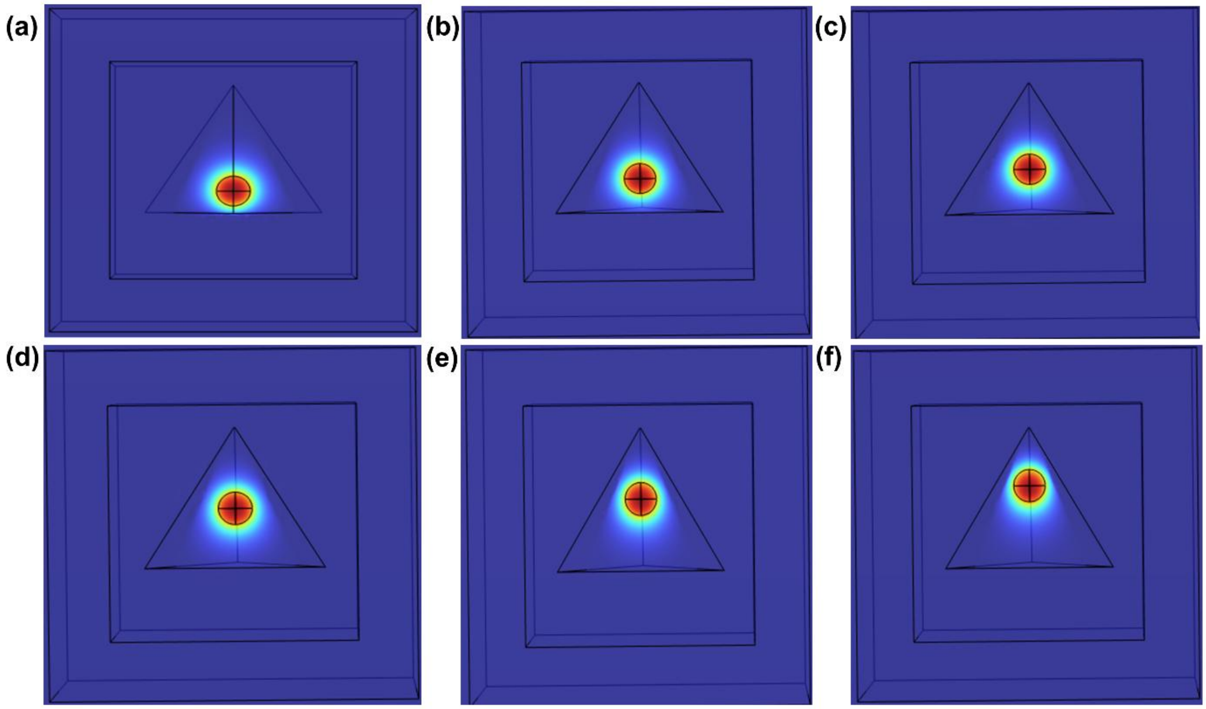

Figure S16. Cross-sectional slices through the center of the cores showcasing the calculated electron wave functions for different core positions (CB offset = 0.4 eV). Panels (a–f) correspond to core positions relative to the centroid of the tetrahedron of -2 nm, -1 nm, 0 nm, 1 nm, 2 nm, and 3 nm, respectively.

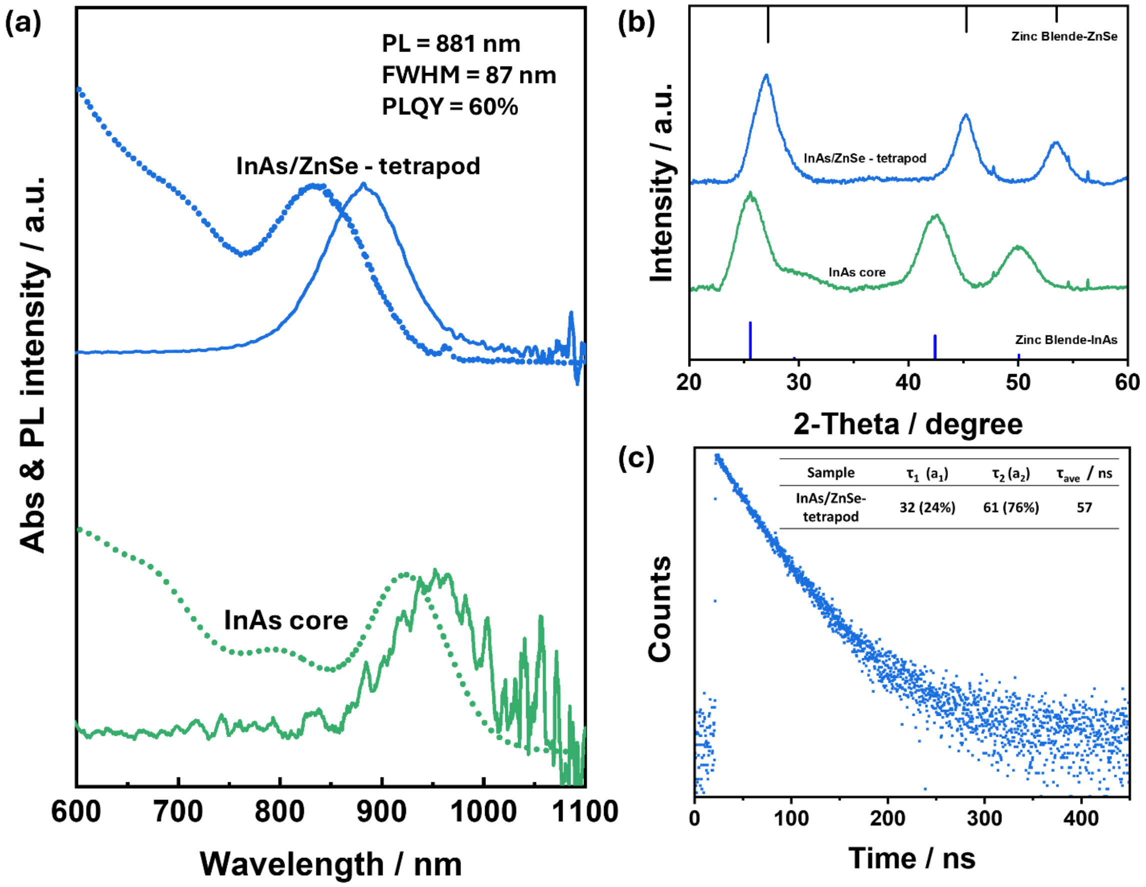

Figure S17. (a) UV–Vis absorption spectra, (b) XRD patterns of InAs core and InAs/ZnSe-tetrapod QDs, (c) PL decay traces of the InAs/ZnSe-tetrapod QDs. The vertcal bars indicate the characteristic peaks of bulk zinc blende InAs (JCPDS No. 15-869). [7]

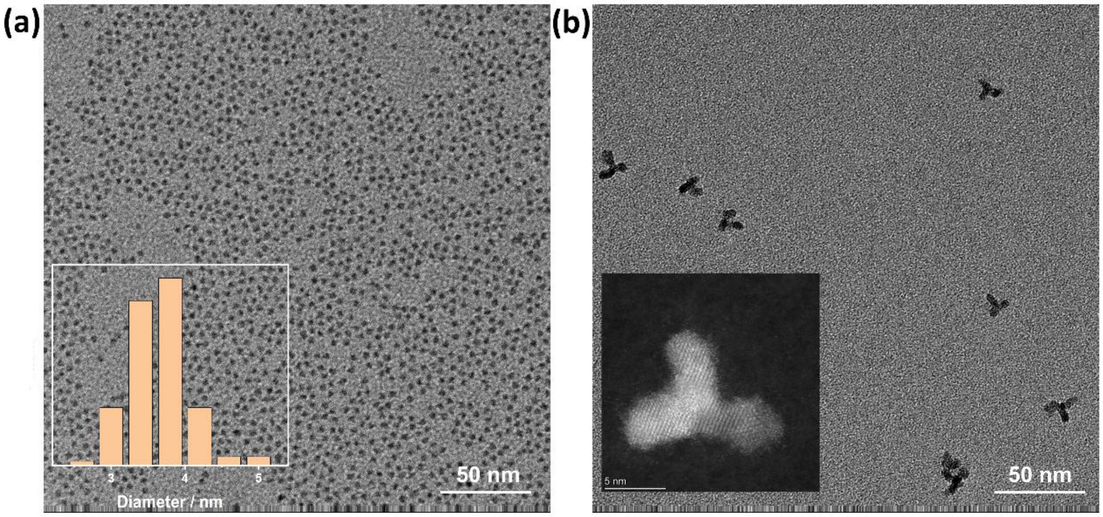

Figure S18. (a) TEM images of InAs core and (b) InAs/ZnSe-tetrapod QDs.

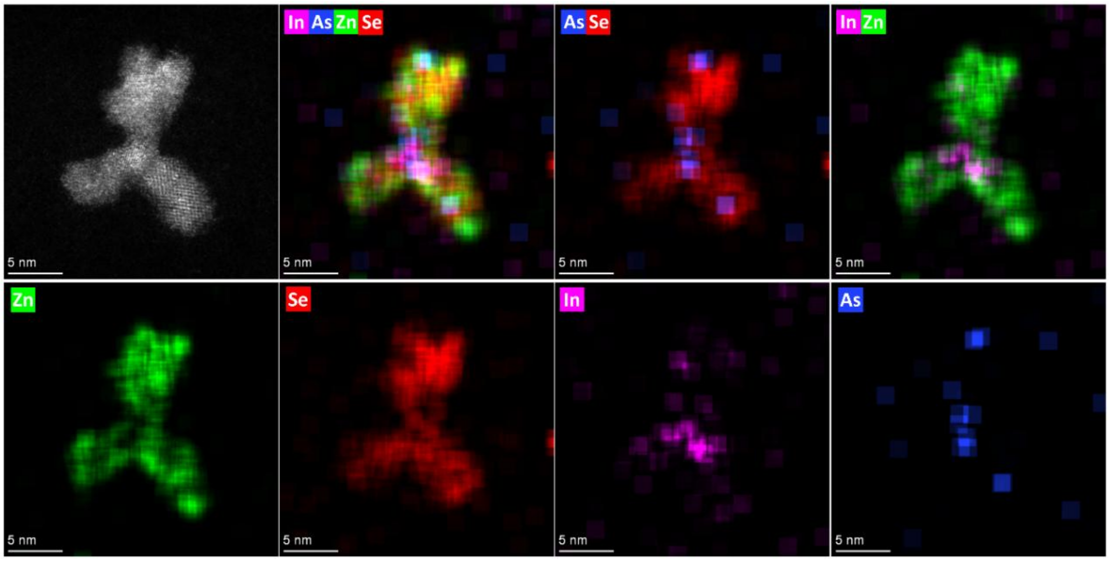

Figure S19. STEM-EDS elemental mapping of the InAs/ZnSe-tetrapod QDs.

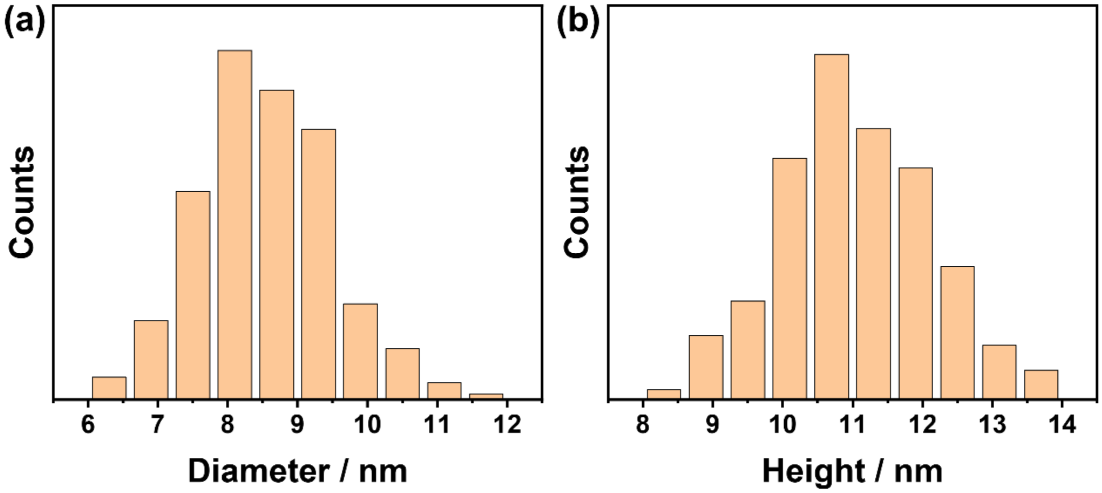

Figure S20. Size distribution histograms and TEM images of the (a)InAs/ZnSe -WZ and (b)InAs/ZnSe -ZB QDs from Figure 5a and 5d.

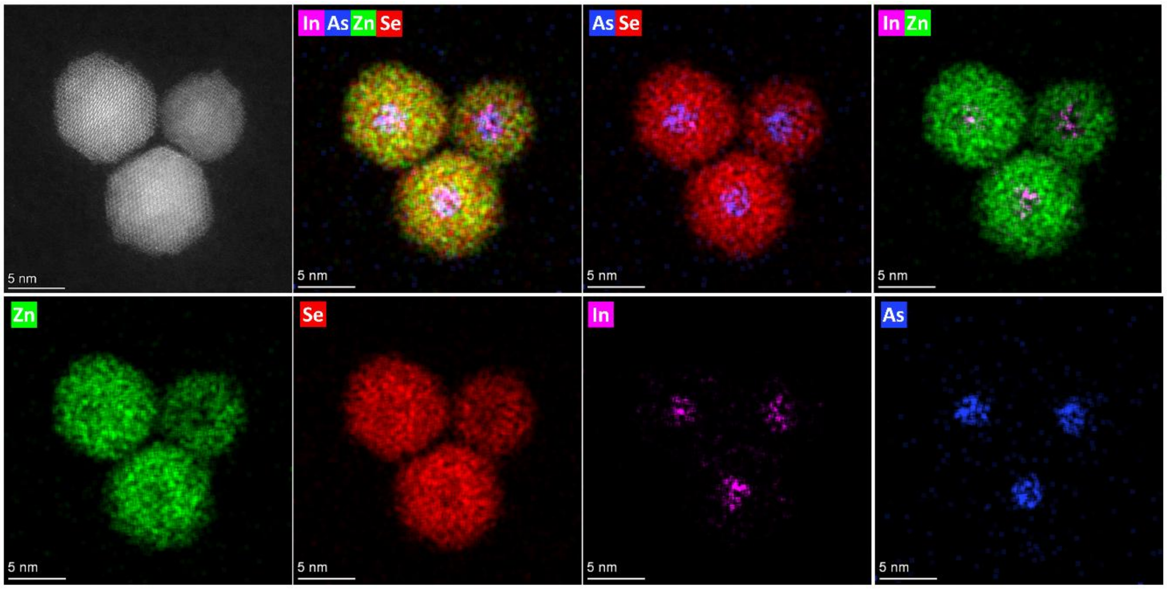

Figure S21. STEM-EDS elemental mapping of the InAs/ZnSe-WZ QDs.

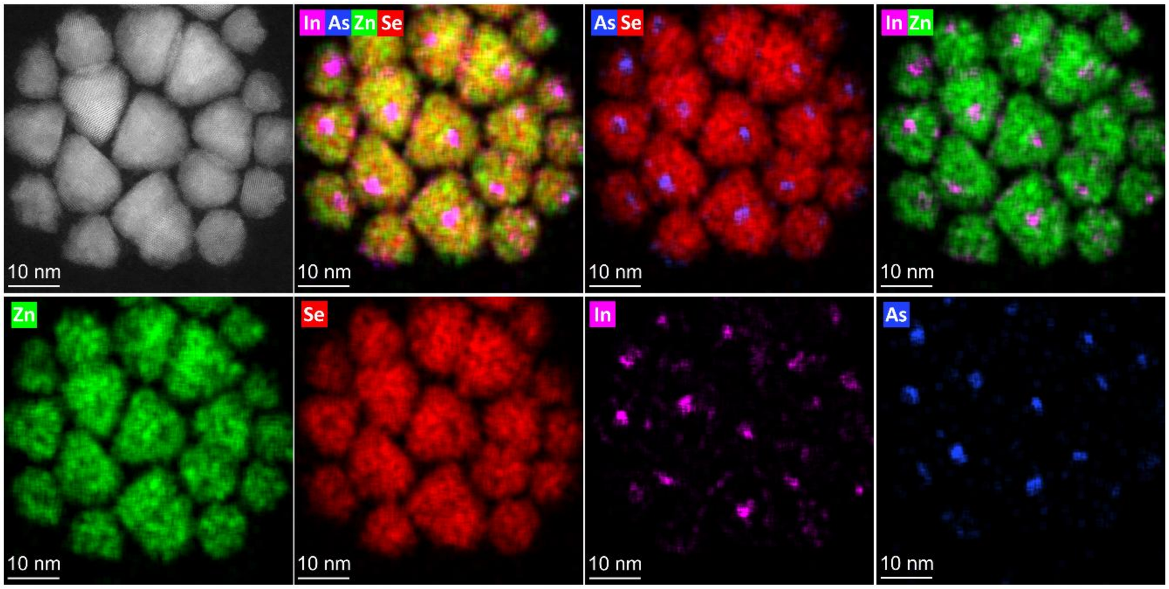

Figure S22. STEM-EDS elemental mapping of the InAs/ZnSe – ZB QDs.

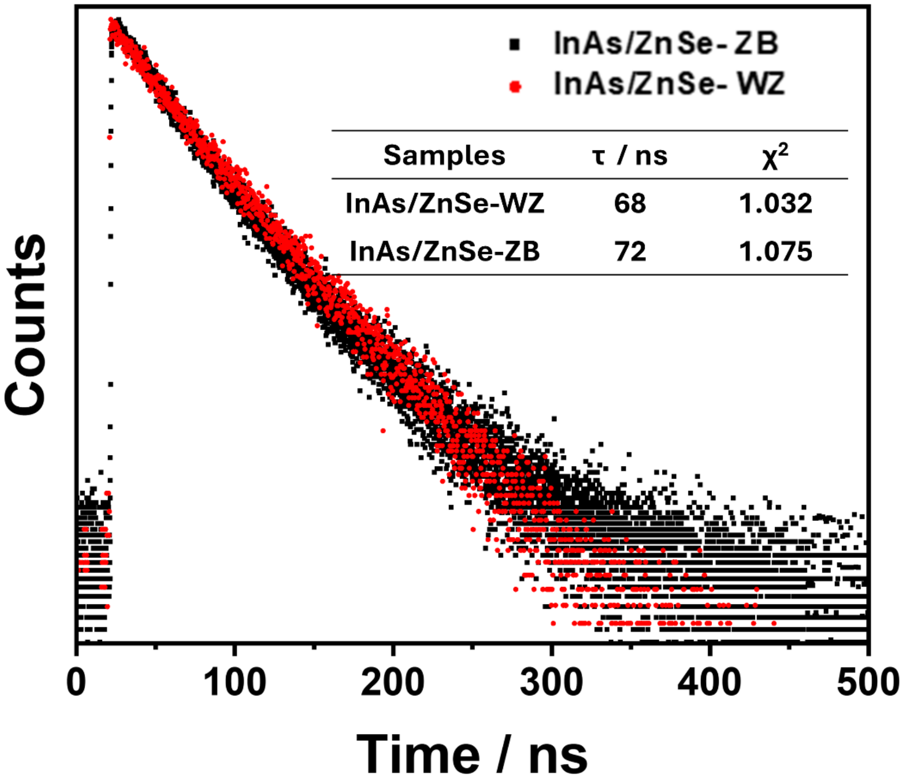

Figure S23. PL decay traces of InAs/ZnSe-ZB and InAs/ZnSe-WZ Q

Table S5. Comparison of our work and the state-of-the-art InAs/ZnSe QDs in terms of PL peak, PLQY, and PL FWHM.

Materials	PL Peak / nm	PLQY / %	PL FWHM / meV	Reference
InAs/ZnSe-WZ	865	90	198	This work
InAs/ZnSe-tetrapod	881	60	139	This work
InAs/ZnSe-ZB	940	81	127	This work
InAs/ZnSe	900	70	208	Adv. Sci. 2024 [8]
InAs/ZnSe	798	N/A	192	Chem. Mater. 2024 [9]
InAs/ZnSe	1020	N/A	156	Adv. Optical Mater. 2024[10]
InAs/ZnSe	825-870	69-72	135-141	J. Am. Chem. Soc. 2024 [11]
InAs/ZnSe	900-947	46-69	182-228	Adv. Mater. 2023 [12]
InAs/ZnSe	860	42	200	J. Am. Chem. Soc. 2022 [13]
InAs/ZnSe	931	43	210	ACS Energy Lett. 2022 [14]
InAs/ZnSe	1029	8	203	Part. Part. Syst. Charact. 2018 [15]
InAs/ZnSe	1010	5-10	180	J. Am. Chem. Soc. 2016 [16]
InAs/ZnSe/ZnS	766-782	25-37	278-321	J. Mater. Chem. C. 2014 [17]
InAs/ZnSe	755	15	257	Chem. Mater. 2010 [18]
InAs/ZnSe	812	7-10	229	J. Am. Chem. Soc. 2006 [19]
InAs/ZnSe	1033	7-20	235	J. Am. Chem. Soc. 2000 [4]

References

[1] H. Lange, D. F. Kelley, *J. Phys. Chem. C*, **2020**, 124, 22839.

[2] O. Madelung, *Semiconductors: data handbook*. Springer Science & Business Media, 2004.

[3] Y. Hinuma, A. Grüneis, G. Kresse, F. Oba, *Phys. Rev. B*, **2014**, 90, 155405.

[4] Y. Cao, U. Banin, *J. Am. Chem. Soc.* **2000**, 122, 9692.

[5] Y. E. Panfil, D. Shamalia, J. Cui, S. Koley, U. Banin, *J. Chem. Phys.* **2019**, 151, 224501.

[6] P. Reiss, M. Protière, L. Li, *Small,* **2009**, 5, 154.

[7] M. Kim, J. Lee, J. Jung, D. Shin, J. Kim, E. Cho, Y. Xing, H. Jeong, S. Park, S. H. Oh, Y. Kim, S. Jeong, *J. Am. Chem. Soc.* **2024**, 146, 10251.

[8] H. Roshan, D. Zhu, D. Piccinotti, J. Dai, M. D. Franco, M. Barelli, M. Prato, L. D. Trizio, L. Manna, F. D. Stasio, *Adv. Sci.* **2024**, 11, 2400734.

[9] D. Mastrippolito, M. Cavallo, H. B. Jalali, G. O. Eren, E. Bossavit, H. Zhang, T. Gemo, A. Colle, A. Khalili, C. Gureghian, Y. Prado, M. G. Silly, D. Pierucci, F. D. Stasio, E. Lhuillier, *Chem. Mater.* **2024**, 36, 11669.

[10] E. Bossavit, O. Yeromina, D. Mastrippolito, M. Cavallo, H. Zhang, T. Gemo, A. Colle, A. Khalili, A. Shcherbakov, L. D. Nguyen, C. Abadie, E. Dandeu, M. G. Silly, B. Gallas, D. Pierucci, A. Degiron, P. Reiss, E. Lhuillier, *Adv. Optical Mater.* **2024**, 12, 2401601.

[11] R. Sun, J. Zang, R. Lai, W. Yang, B. Ji, *J. Am. Chem. Soc.* **2024**, 146, 17618.

[12] D. Zhu, H. B. Jalali, G. Saleh, F. D. Stasio, M. Prato, N. Polykarpou, A. Othonos, S. Christodoulou, Y. P. Ivanov, G. Divitini, I. Infante, L. D. Trizio, L. Manna, *Adv. Mater.* **2023**, 35, 2303621.

[13] D. Zhu, F. Bellato, H. B.Jalali, F. D. Stasio, M. Prato, Y. P. Ivanov, G. Divitini, I. Infante, L. D. Trizio, L. Manna, *J. Am. Chem. Soc.* **2022**, 144, 10515.

[14] M. D. Franco, D. Zhu, A. Asaithambi, M. Prato, E. Charalampous, S. Christodoulou, I. Kriegel, L. D. Trizio, L. Manna, H. B. Jalali, F. D. Stasio, *ACS Energy Lett.* **2022**, 7, 3788.

[15] R. Tietze, R. Panzer, T. Starzynski, C. Guhrenz, F. Frenzel, C. Würth, U. R. Genger, J. J. Weigand, A. Eychmüller, *Part. Part. Syst. Charact.* **2018**, 35, 1800175.

[16] V. Grigel, D. Dupont, K. D. Nolf, Z. Hens, M. D. Tessier, *J. Am. Chem. Soc.* **2016**, 138, 13485.

[17] J. Zhang, R. Li, W. Sun, Q. Wang, X. Miao, D. Zhang, *J. Mater. Chem. C*, **2014**, 2, 4442.

[18] J. Zhang, D. Zhang, *Chem. Mater.* **2010**, 22, 1579.

[19] J. P. Zimmer, S. Kim, S. Ohnishi, E. Tanaka, J. V. Frangioni, M. G. Bawendi, *J. Am. Chem. Soc.* **2006**, 128, 2526.
